# Immediate or delayed initiation of renal replacement therapy in patients with leptospirosis and acute kidney injury: a target trial emulation

**DOI:** 10.1186/s13613-025-01477-5

**Published:** 2025-05-14

**Authors:** Marie Julien, Cédric Rafat, Loïc Raffray, Henri Vacher-Coponat, Nicolas Allou, Jérôme Allyn, Julien Jabot, Yannis Lombardi

**Affiliations:** 1 Nephrology Unit, Centre Hospitalier Universitaire Felix Guyon, Saint-Denis, La Réunion France; 2 Renal Intensive Care Unit, Tenon Hospital, Assistance Publique-Hôpitaux de Paris, Paris, France; 3 Department of Internal Medicine, Félix-Guyon University Hospital of La Réunion, CS11021 Saint Denis, La Réunion France; 4 Unité Mixte de Recherche Processus Infectieux en Milieu Insulaire Tropical (UMR PIMIT), Université de La Réunion, CNRS 9192, INSERM 1187, IRD 249, Saint-Denis, La Réunion France; 5 Intensive Care Unit, Centre Hospitalier Universitaire, Saint-Denis, La Réunion France; 6 Institut des Sciences du Calcul et des Données (ISCD), Sorbonne University, Paris, France; 7 Sentinelles Network, Pierre Louis Institute for Epidemiology and Public Health, INSERM and Sorbonne University, 27, Rue Chaligny, BC 2908, 75571 Paris, Cedex 12, France

## Abstract

**Background:**

Anecdotal evidence suggests that early renal replacement therapy (RRT) may improve the mortality associated with acute kidney injury (AKI) in patients with leptospirosis. Conversely, several randomized controlled trials (RCTs) conducted in intensive care units have refuted the positive impact of early RRT on mortality in patients with AKI and other causes of sepsis.

**Methods:**

In this emulated RCT utilizing a propensity score-weighted logistic regression performed in the two academic centers on the island of La Réunion, France, between 2010 and 2020, we evaluated the impact of the timing of RRT on a composite outcome of mortality or new-onset or worsening chronic kidney disease (CKD) within a year, in patients hospitalized with leptospirosis, Stage 3 AKI, and no immediate need for RRT.

**Results:**

We included 295 consecutive patients with leptospirosis and Stage 3 AKI: 82 (28%) began RRT within 48 h of admission (“early” group), 213 (72%) did not start RRT within 48 h (“delayed” group). In the delayed group, 53/213 (25%) patients eventually required RRT. 59/295 patients (20%) met the primary outcome: 32 (15%) in the delayed group and 27 (33%) in the early group. The odds ratio (OR) for primary outcome occurrence before weighing was 2.78 (95% confidence interval CI 1.53 to 5.01, p < 0.001; reference: delayed group) and after weighting was 2.08 (95% CI: 1.01 to 4.26, p = 0.046). In secondary analyses, there was a significantly higher probability of CKD occurrence in the early group (OR 2.74, 95% CI 1.25 to 6.0, p = 0.012). Mortality at 1 year did not differ between groups (OR 0.76, 95% CI 0.21 to 2.68, p = 0.666).

**Conclusion:**

Early initiation of RRT may be associated with an increased risk of death and development of CKD within 1 year in patients with leptospirosis and Stage 3 AKI.

**Supplementary Information:**

The online version contains supplementary material available at 10.1186/s13613-025-01477-5.

## Introduction

Leptospirosis, a zoonotic and waterborne disease caused by *Leptospira spp*, poses a global public health challenge [1, 2]. Its incidence has exhibited a steady increase over the years, ascribed in part to global warming and climate change. This trend has resulted in an annual toll of over 60,000 deaths [2].

Acute kidney injury (AKI) stands out as a hallmark of the disease, serving as a defining feature of febrile hepato-renal syndromes in tropical regions across the globe [3–5]. In 2019, on the island of La Réunion, more than half of the patients with reported leptospirosis developed AKI [6]. Renal involvement in leptospirosis ranges from asymptomatic urinary abnormalities to severe AKI requiring renal replacement therapy (RRT). The most common form of renal manifestation is acute tubulointerstitial nephritis [7, 8].

Leptospirosis-induced AKI (AKI-L) is typically non-oliguric in the initial stage and often associated with hypokalemia and sodium wasting due to tubular defects [9–12]. The physiopathology of AKI-L involves hypotension, hypovolemia, inflammation, rhabdomyolysis, bile acid toxicity and the detrimental effects of leptospiral proteins on the outer membrane proteins [8, 13–16].

Leptospirosis may cause kidney fibrosis, culminating in chronic kidney disease (CKD-L) because of tubulointerstitial nephritis, the persistence of leptospires in renal proximal tubules and the activation of pro-inflammatory pathways [8, 17–20]. CKD-L has been proposed as a potential contributor to chronic kidney disease of unknown etiology (CKDu), which is increasingly acknowledged as a significant burden in tropical rural settings [21, 22].

A single study in 2007 investigated the impact of the timing of RRT initiation in the course of AKI-L and suggested a benefit on mortality with the implementation of an early and daily RRT strategy [23], with the rationale that such a regimen would minimize uremic complications and improve outcomes in this specific population, typically free of comorbidities. Nonetheless, these results are at odds with several landmark trials performed in the intensive care settings on patients with septic shock, which found no benefit from early compared with delayed RRT, on patient mortality and other outcomes [24–26].

In light of these conflicting findings, the optimal timing for initiating RRT in leptospirosis patients with AKI remains unclear. A pragmatic randomized controlled trial (RCT) comparing early and delayed RRT initiation would provide the highest level of evidence, but such an approach is beset by multiple challenges, primarily patient enrolment. Alternatively, appropriately designed target trial emulation studies, which utilize observational data to simulate a hypothetical RCT, can offer a robust alternative if an RCT is not feasible [27–29].

In this study, our objective was to assess the impact of the timing of RRT initiation on mortality and kidney function (including new-onset or worsening of CKD) up to one year post-hospital discharge. We utilized an emulated RCT approach in patients hospitalized with leptospirosis, specifically those presenting with Stage 3 AKI upon admission and no immediate requirement for RRT.

## Methods

### Study design

Our main objective was to determine whether there is a causal effect of the timing of RRT initiation on mortality and kidney function in patients with leptospirosis and severe AKI. To address this question, we emulated a target RCT using data from an observational cohort study.

### Observational cohort study

We conducted a retrospective cohort study in two academic centers—Saint-Denis and Saint-Pierre hospitals—on the island of La Réunion, France. The study received approval from the ethics committee of the French Intensive Care Society (#CE SRLF20-37), which waived the requirement for individual informed consent in accordance with French policies.

Screening consisted of all adult patients with at least one inpatient visit between January 1, 2010, and December 31, 2020. All consecutive patients with at least one International Classification of Disease, 10 th edition code A27 (“Leptospirosis”) in the hospitals’ administrative database were targeted.

A single physician reviewed all the electronic health records of the targeted patients (MJ). Patients were included in the cohort study if they met both of the following criteria during the same inpatient visit:

1. Documented leptospirosis, defined as at least one of the following:
  - Positive urine and/or blood polymerase chain reaction detecting pathogenic *Leptospira* using a portion of ribosomal RNA 23S as target
  - Positive serology using the microscopic agglutination test of Martin and Pettit (performed by the National Reference Center of the Pasteur Institute, Paris) with antibody titers greater than 1:400 and/or a positive enzyme-linked immunosorbent assay (SERION) with IgM antibody levels higher than 50 UI/L [4, 30]
2. Stage 2 or 3 AKI upon hospital entry, according to the criteria of the Kidney Disease Improving Global Outcome (KDIGO) [31].

Baseline characteristics, outcomes during hospitalization and follow-up information up to 1 year after hospital discharge were collected by the same physician (MJ) using electronic health records. Mortality and CKD data were assessed after hospital discharge and at one year by reviewing the electronic health record if the patient returned to the hospital (for a post-hospitalization follow-up consultation or other reason) or by calling the patient or treating physician in the opposite case.

### Target trial

The “emulation of a target trial” framework requires the explicit definition of a “target trial”, which represents the ideal RCT one would hypothetically conduct to optimally address the appointed research question. The design of our target trial (Table S1) was adapted from the Artificial Kidney Initiation in Kidney Injury (AKIKI) trial design [24]. In summary, our target trial fulfilled the following specifications:

- Inclusion: documented leptospirosis with Stage 3 AKI upon hospital admission
- Treatment strategies: early initiation of RRT (i.e., immediately following randomization, and mandatorily within 48 h following hospital entry); or delayed initiation of RRT (i.e., randomization, and subsequent observation with initiation of RRT if—and *only* if—a pre-specified criterion for severity was met)
- Outcome: composite outcome assessed at one year: death or new-onset or worsening CKD.

The latter was used to estimate the incidence of CKD-L during follow-up; the definition of new-onset or worsening CKD was similar to that used in a post-hoc analysis of the AKIKI cohort [32] (Table S2).

### Observational analogue

The observational analogue was specified so that its design was as close as possible to the target trial. The assessment of the quality of the observational analogue was performed following a previously validated strategy (Table S3) [27].

### Main analysis

The steps followed to emulate the target trial are detailed in the Supplemental Methods and summarized as follows:

1. Allocation: patients were assigned either to the “early RRT” group (RRT initiated within 48 h) or the “delayed RRT” group (RRT *not* initiated within 48 h),
2. Outcome adjudication: the main outcome was met if the patient had died or developed new-onset or worsening CKD within a year,
3. Imputation for missing data: missing data were imputed using multiple imputations,
4. Derivation of a propensity score: in each imputed dataset, a propensity score estimating the probability of being assigned to the early RRT group was derived, using data available at the time of hospital admission; variables included in the score were those used in the SAPSII score plus baseline eGFR, which are confounding factors (i.e., linked both to outcome occurrence and RRT initiation),
5. Estimation of the treatment effect: in each dataset, the weighted odds ratio (OR) for outcome occurrence according to the treatment strategy was determined using inverse probability of treatment weighting,
6. Summary of the treatment effect: weighted ORs were pooled across datasets,
7. Assessment of the balance between groups: standardized mean differences and variances between groups regarding baseline covariates were assessed, before and after weighting.

### Sensitivity analyses

Sensitivity analyses were performed to test the robustness of the results obtained in the main analysis. These sensitivity analyses assessed the impact of covariates, functional forms, the method used for weighting, the impact of the exclusion strategy, and the impact regarding the data management of the two patients who died within 48 h. In each sensitivity analysis, exactly the same steps as in the main analysis were repeated, except that a specific parameter was modified.

### Subgroup and secondary analyses

In subgroup analyses, we replicated the same steps as in the main analysis but focused on different population subsets. In a secondary analysis, we performed the same steps as in the main analysis but used each individual component of the main outcome for the main analysis.

### Statistical software

All analyses were performed using R version 4. All tests are two-sided and a p-value < 0.05 is considered significant.

### Reporting

Results are reported following the guidelines of the Strengthening the Reporting of Observational Studies in Epidemiology initiative [33]. The checklist is available in the Supplementary File.

## Results

### Characteristics and outcomes in the source population

Over the study period, 654 patients were admitted for documented leptospirosis in the two academic centers of La Réunion. 380 (58%) of these patients developed AKI Stage KDIGO 2 or 3 and were subsequently included in our cohort study. Detailed patients’ baseline characteristics and outcomes in the cohort study are detailed in Table S4.

Patients were predominantly men (94%), young (median age 51 [41–59] years) and had few associated conditions: 23% had arterial hypertension, 18% diabetes mellitus and 7% had CKD at baseline. On hospital admission, the most common clinical symptoms included fever (95%), myalgia (83%), jaundice (70%) and oligo/anuria (58%). Blood platelet count was below 30 G/L in 166 (44%) patients. Median creatinine was 410 μmol/L (interquartile range [IQR]: 301 to 550) and median urea was 20 mmol/L (IQR: 15 to 26).

Patients were severely ill: 83% had KDIGO 3 AKI upon hospital entry, 71% required an intensive care unit (ICU) stay, and 18% required invasive ventilation. All patients were treated with antibiotics for a median of 7 days. First-line antibiotic therapy consisted of amoxicillin in 64% of cases; second line in third generation cephalosporins in 13% of cases. Other antibiotics used were macrolides, doxycycline, quinolones and amoxicillin-clavulanic acid.

### Trial emulation

**Fig. 1:**
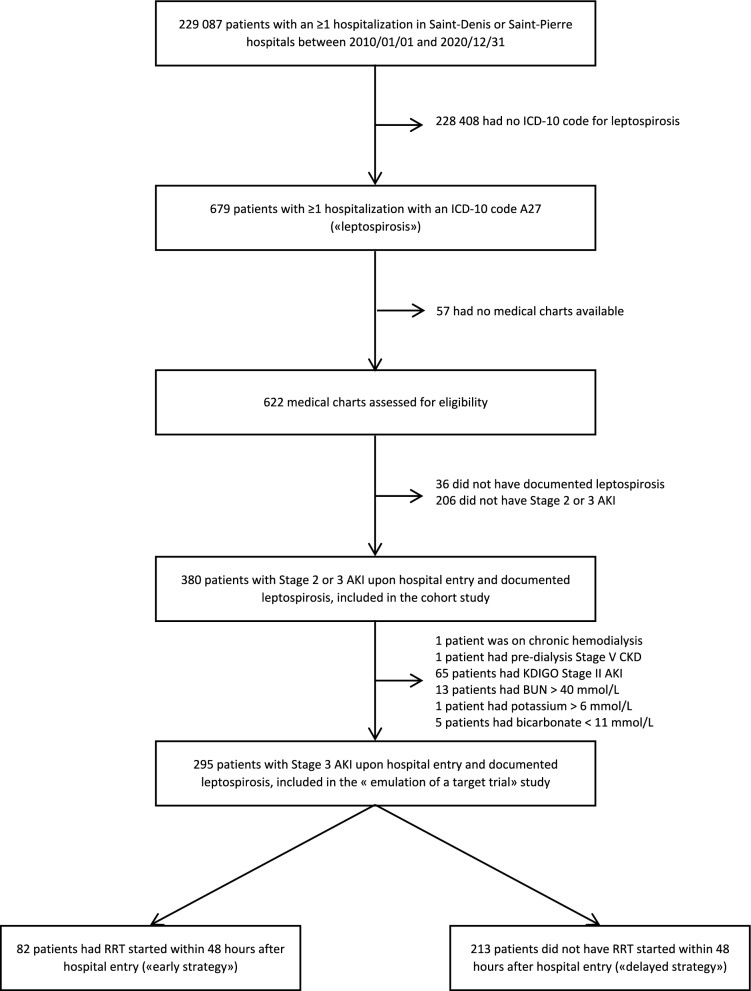
Flow chart of patients’ selection process. Multiple reasons for exclusion can apply for a single patient. *AKI* acute kidney injury, *BUN* blood urea nitrogen, *CKD* chronic kidney disease, *ICD-10* International Classification of Disease, 10 th edition, *KDIGO* Kidney Diseases Improving Global Outcomes, *RRT* renal replacement therapy

Patient selection process for the emulated trial is described in Fig. 1 and baseline characteristics before and after propensity-score weighting are described in Tables 1 and S5.

**Table 1:** Values at baseline of the covariates used in the propensity score according to the treatment strategy, before and after propensity-score weighting

Variable	Before weighting			After weighting*		
	Delayed strategy (n = 213)	Early strategy (n = 82)	SMD %	Delayed strategy (n = 213)	Early strategy (n = 82)	SMD %
Demography and medical history						
Age, years	51.0 (42.0, 59.0)	52.0 (38.0, 60.0)	**11**	51.0 (41.0, 60.0)	53.0 (37.7, 62.0)	2
Cancer	9.0 (4.2%)	2.0 (2.4%)	**10**	11.4 (4.0%)	9.1 (3.0%)	5
Baseline eGFR, ml/min/1.73 m^2^	103.2 (90.8, 109.6)	101.0 (86.2, 111.8)	3	103.3 (90.9, 109.9)	100.4 (86.7, 112.2)	3
Physiology						
Heart rate, bpm	96.0 (85.0, 110.0)	106.0 (93.5, 119.0)	**34**	99.0 (86.0, 112.0)	105.0 (94.0, 114.3)	**17**
Systolic blood pressure, mmHg	120.0 (107.0, 132.8)	110.0 (90.0, 130.0)	**50**	119.9 (104.0, 131.0)	120.8 (100.0, 140.9)	5
Temperature, °C	37.3 (36.7, 38.2)	37.1 (36.3, 38.1)	**24**	37.3 (36.7, 38.2)	37.5 (36.7, 38.1)	4
Glasgow coma scale < 15	5.0 (2.3%)	6.0 (7.3%)	**23**	10.6 (3.7%)	11.7 (3.9%)	1
Biology						
Arterial partial O^2^ pressure, mmHg	83.5 (71.3, 96.0)	88.0 (77.0, 101.0)	**30**	84.0 (72.6, 98.0)	86.6 (78.0, 95.0)	1
Urea nitrogen, mmol/L	18.7 (13.9, 24.6)	22.8 (17.2, 30.3)	**52**	19.9 (14.6, 25.4)	20.0 (16.7, 24.8)	9
Potassium, mmol/L	3.5 (3.2, 3.8)	3.7 (3.3, 4.1)	**33**	3.6 (3.2, 3.9)	3.6 (3.2, 3.9)	5
Bicarbonates, mmol/L	22.0 (20.0, 24.0)	20.0 (17.0, 22.0)	**62**	22.0 (20.0, 24.0)	22.0 (19.0, 25.0)	3
Sodium, mmol/L	133.0 (130.0, 136.0)	132.0 (130.0, 135.0)	**10**	133.0 (129.0, 136.0)	132.0 (129.0, 134.9)	**17**
Total bilirubin, µmol/L	114.0 (43.3, 202.3)	154.0 (72.0, 252.0)	**36**	128.0 (48.8, 241.0)	152.6 (68.8, 219.2)	8
Leucocytes, 10^9^ cells/L	10.2 (8.2, 13.3)	11.4 (8.4, 14.6)	**29**	10.3 (8.2, 13.5)	9.9 (7.6, 14.1)	1

*Bpm beats per minute, eGFR estimated glomerular filtration rate, SMD absolute standardized mean difference (mean across all 100 imputed datasets). Standardized mean differences above 10% are indicated in bold.*

^*^For illustrative purposes, summary statistics from the first (of 100) imputed datasets are shown

Two hundred and ninety-five patients were included in the emulated trial. Eighty-two (27.8%) patients received RRT within 48 h of hospital admission and were assigned to the early group. Two hundred and thirteen patients (72.2%) did not receive RRT within 48 h and were assigned to the delayed group: in this group, 53/213 (24.9%) eventually underwent RRT at a later stage. In the delayed cohort, mortality at one year was higher in patients who ultimately required RRT (6/53 (11%) versus 3/160 (2%), p = 0.008 for comparison using Fisher’s test). The median time to RRT initiation was 2 days (IQR: 1 to 3).

**Fig. 2:**
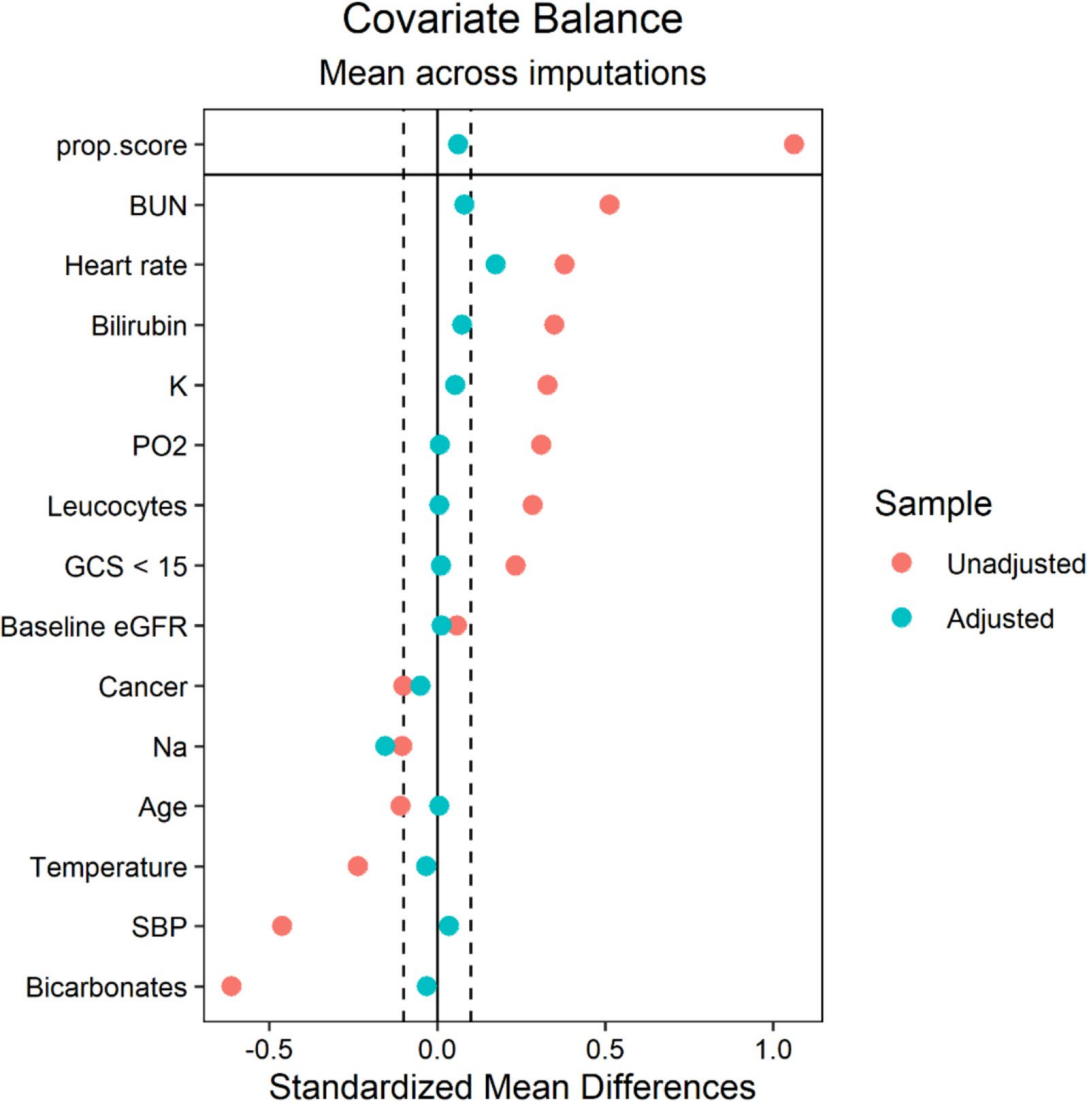
Standardized mean differences between groups for covariates included the propensity score before and after weighting. *BUN* blood urea nitrogen, *GCS* Glasgow coma scale, *eGFR* estimated glomerular filtration rate, *K* potassium, *Na* sodium, *PO2* partial arterial oxygen pressure, *SBP*: systolic blood pressure. A standardized mean difference (SMD) > 0 for a given variable indicates a higher value in the “early RRT” group compared to the “delayed EER” group. Balance is achieved for a given variable if SMD is between −10% and 10% (i.e., between the dotted lines)

Fifty-nine (20%) patients met the primary outcome (Tables 2 and S6): 12/295 (4.1%) died during their hospital stay, 2/295 (0.7%) were discharged alive and died within a year, 45/295 (15.3%) were alive and developed new-onset or worsening CKD at one year. Patients classified with new-onset or worsening CKD exhibited CKD Stage III in a majority of cases (32/45). A significant minority of patients displayed severe CKD upon follow-up: 7/45 had CKD Stage IV and 3/45 CKD Stage V. Before adjustment, balance was achieved for 1/14 (7.1%) of the covariates included in the propensity score (Figs. 2 and S1). This indicates substantial in-between-group differences for these covariates collected upon hospital admission. The differences systematically showed greater severity in the early compared to the delayed group, prompting propensity-score weighting. Balance was subsequently achieved for 12/14 (85.7%) of the covariates included in the propensity score (Figs. 2 and S1).

**Table 2:** Outcomes according to the treatment strategy

	Delayed strategy (n = 213)	Early strategy (n = 82)	Before weighting		After weighting	
			OR (95% CI)	p	OR (95% CI)	p
Primary outcome						
Death or new-onset or worsening CKD at 1 year	32 (15%)	27 (33%)	2.78 (1.53 to 5.04)	** < 0.001**	2.08 (1.01 to 4.26)	**0.046**
Secondary outcomes						
Death within a year	9 (4.2%)	5 (6.1%)	1.47 (0.47 to 4.57)	0.502	0.76 (0.21 to 2.68)	0.666
New-onset or worsening CKD at one year						
Alive and with new-onset or worsening CKD	23 (11%)	22 (27%)	3.03 (1.57 to 5.85)	**0.001**	2.74 (1.25 to 6.00)	**0.012**
New-onset or worsening CKD, excluding patients who died	23 (11%)	22 (29%)	3.15 (1.62 to 6.11)	**0.001**	2.40 (1.07 to 5.35)	**0.033**

*CI confidence interval, OR odds ratio, CKD chronic kidney disease. p-values < 0.05 are indicated in bold.*

In the main analysis (Table 2), the adjusted OR for primary outcome occurrence was 2.08 (95% confidence interval: 1.01 to 4.26, p = 0.046; reference group: delayed strategy). Twenty-four sensitivity analyses were performed to assess the robustness of these findings under various hypotheses (Table S6, Figure S2). 15/24 (63%) sensitivity analyses found results similar to the main analysis. No analysis found a lower incidence of the primary outcome in patients in the early strategy. In 9/24 (38%) analyses, no statistically significant difference between groups was detected, under the following conditions: i) Weighting using baseline Simplified Acute Physiology Score (SAPS) II score, Sequential Organ Failure Assessment (SOFA) score, or a combination of both; ii) initial respiratory severity assessed using “dyspnea” instead of “arterial PO2”; iii) Inclusion of creatine kinase, or of SAPSII score, SOFA score, or a combination of both, in the variables used for weighting; and iv) exclusion of patients with arterial partial pressure of carbon dioxide (PCO2) > 50 mmHg at baseline.

In subgroup analyses (Table S7), there was a significantly higher incidence of the primary outcome in the early strategy group, within the subgroup of patients with no pre-existing CKD. There was no significant difference between strategies in subgroups defined by age or gender.

In secondary analyses (Table 2), there was a significantly higher probability of new-onset or worsening CKD at one year in patients assigned to the early strategy. There was no significant difference regarding the probability of death at one year.

In an exploratory univariate analysis performed among the variables included in the propensity score, the two main factors associated with being alive and with a new-onset or worsening CKD at one year were baseline eGFR and blood urea nitrogen upon hospital entry (Table S8). Preexisting hypertension or diabetes mellitus were not associated with the occurrence of new-onset or worsening CKD (p = 0.622 and 0.415, respectively).

In another exploratory analysis, we compared the reasons for RRT initiation and outcome occurrence between patients in the early group and those in the delayed group who eventually required RRT. Using values collected on the day of RRT initiation, we found that in the early strategy, 53/82 patients (64%) had none of hyperkalemia, BUN > 30 mmol/L, or bicarbonate ≤ 11 mmol/L, compared to 13/53 patients (25%) in the delayed group (p < 0.001). In terms of outcomes, 5/82 patients (6%) in the early strategy died and 22/77 (29%) had new-onset or worsening CKD, compared to 6/53 patients (11%) and 11/42 (26%) in the delayed group, respectively (p = 0.34 and p = 0.83).

## Discussion

### Key results

In our emulation of a RCT involving 295 patients with leptospirosis and KDIGO Stage 3 AKI, early initiation of RRT was not found to improve the combined outcome of death or development of new-onset or worsening CKD at one year. In the delayed strategy cohort, RRT was ultimately required in only 25% of patients. Across all the secondary, sensitivity and subgroup analyses, the results consistently demonstrated that an early initiation of RRT was not associated with improved outcomes. These results advocate against an early initiation of RRT in patients with leptospirosis and severe AKI outside the conventional framework and set of emergent criteria determined by large randomized studies [24–26].

To the best of our knowledge, our study of 380 patients recruited over 11 years is the largest retrospective study focused specifically on leptospirosis with severe AKI. Our results highlight the persisting severity of leptospirosis, even in young patients with few associated conditions – as evidenced by a mortality rate of 5% in our cohort, with a substantial proportion of patients (39%) requiring RRT. We also found that a sizeable proportion of patients developed new-onset or worsening CKD (15.3%), most likely due to CKD-L.

### Interpretation and generalizability

These findings are in contrast to those of the landmark Brazilian study by Andrade et al. [23], which argued in favor of an interventionist RRT strategy. This study compared an early daily RRT strategy using intermittent hemodialysis (IHD) or sustained low-efficiency dialysis with a delayed tri-weekly RRT strategy, in 33 patients admitted to the ICU. Mortality was reduced in the early and daily RRT group (16.7% versus 66.7%), suggesting that early initiation dialysis and high dialysis dose may significantly improve survival in patients with severe leptospirosis-associated AKI. The study was underpinned by a sound pathophysiological rationale, addressing key challenges in leptospirosis such as enhanced fluid management, improved control of azotemia, and mitigation of hemorrhagic complications [8, 11, 34]. However, the study's design (before/after) is inherently prone to bias [35], and the unadjusted analysis, small single-center cohort (n = 33), and limited number of events restrict the generalizability of these results. Despite the potential limitations, these findings have served as a foundation for numerous guidelines or reviews recommending an early initiation of RRT in patients with leptospirosis. These recommendations have held true both in the pre-AKIKI [36–38] and post-AKIKI [4, 8, 39–41] eras.

Our results do not support any benefit on mortality or kidney-related outcomes of an early RRT initiation in patients without an urgent need for RRT upon hospital admission. This effect was observed consistently across the main analysis and all the 24 subsequent sensitivity analyses. In summary, our study’s conclusion aligns with that of the AKIKI [24], STARRT-AKI [25] and IDEAL ICU [26] trials, conducted in predominantly, or exclusively, septic critically ill patients.

In addition, our study results suggest a higher incidence of adverse renal outcomes with early initiation of RRT. These results may stem from a genuine treatment effect ascribed to hemodynamic instability related to renal replacement therapy (HIRRT). HIRRT refers to the complex interplay of factors whereby RRT may inadvertently sustain RRT-dependence through a negative feedback loop involving recurring hemodynamic instability [24, 42, 43].

Alternatively, the fact that several sensitivity analyses using alternative confounding variables found no statistically significant difference between groups is indicative of residual confounding. Consequently, our study does not provide a definitive answer to whether a delayed strategy leads to improved kidney outcomes compared to an early approach. Seventy-five percent of patients in the delayed RRT group ultimately did not require RRT, exceeding the ratio reported in the AKIKI trial [24]. Hypovolemia and hypokalemia associated with digestive losses and tubular dysfunction specific to leptospirosis may partly account for these results [8, 11, 12, 15, 34]. A significant fraction of patients with AKI-L may in fact be responsive to volume repletion strategies while the combination of low potassium levels and maintained urine output may delay RRT initiation. It should be noted that, since the clinical course of leptospirosis is characterized by a multi-stage evolution, with the onset of AKI initially progressing slowly and then accelerating—coinciding with the emergence of leptospirosis-specific antibodies and the detection of leptospires in the urine—it is possible that admitted patients had progressively developed Stage 3 AKI in the days preceding hospitalization.

This delayed RRT strategy, which allows spontaneous recovery of kidney function thereby avoiding the need for RRT, may hold significant medico-economic significance in resource-limited regions where leptospirosis is often endemic [2, 44]. Finally, this conservative approach may also minimize the risk of iatrogenic complications associated with catheter placement, especially in the setting of severe thrombocytopenia and increased hemorrhagic risk associated with leptospirosis. It is noteworthy that patients in the delayed strategy group who ultimately required late RRT exhibited a higher mortality than those who did not (11% versus 2%, p = 0.008), a pattern reminiscent of the data described in the AKIKI trial [24].

Leptospirosis has been considered as one of the potential culprits behind CKDu, perplexing both investigators in Sri Lanka—a tropical island in the Indian Ocean—and in the Mesoamerican region [45, 46]. Investigators have previously found a heightened Leptospira-associated seroprevalence among patients with CKDu compared with controls. Limited data exists on leptospirosis follow-up with respect to kidney function. Nevertheless, our incidence of 15.3% patients with CKD-L aligns with that of previous cohorts [47, 48]. Inconsistent follow-up and the absence of kidney pathology restricts the significance of our findings. In addition, it may be argued that new-onset or worsening CKD may merely reflect the transition of AKI to CKD which is closely associated with the severity of the initial kidney insult, especially when RRT is required [49]. However, in our cohort the incidence of severe CKD-L (defined by CKD Stage III or more) was 16%, highlighting leptospirosis as a potential contributing factor for CKD. Furthermore, the occurrence of CKD-L was not found to be influenced by traditional preexisting kidney risk factors (e.g., diabetes, hypertension) for non-kidney function recovery, suggesting that leptospirosis per se may play a role in the genesis of CKD.

### Strengths and limitations

We conducted a large emulation of an RCT study, using a framework which is increasingly considered a standard for observational studies that investigate the effect of interventions [28]. The specifications of our observational analogue met the criteria for achieving a close emulation of the target trial [27], thus attaining the highest level of evidence, outside an RCT. We used a systematic procedure to include all consecutive patients in the two academic hospitals authorized to provide care for critically ill patients in La Réunion over a considerable period. Because nearly all eligible patients within our specified geographical area were included, our study minimized biases and can be considered population-based across the study's geographic region. Additionally, immortal time bias was accounted for in our study.

Our study displays several limitations. The comparison of our study results with those of Andrade was constrained by differences in RRT modalities between the two studies, with our study predominantly utilizing continuous techniques, in contrast to the intermittent technique employed in the Brazilian study. Furthermore, part of the beneficial impact of the hemodialysis protocol may lie in the increased dose, rather than in the timing of initiation, and this finding could remain valid were an intermittent technique to be selected to treat AKI-L. Second, given that our study was conducted in a geographic area in which medical and economic resources meet high resources standards, the results might not apply to other settings with more restricted access to RRT. Third, given the non-randomized nature of our study, residual confounding—which could change the study’s conclusion—cannot be ruled out. However, this seems unlikely, since across all 24 sensitivity analyses, which accounted for confounding in several ways, the same result was consistently observed (i.e., no beneficial effect of early RRT). Fourth, the decision to initiate RRT in the “delayed strategy” was not performed as per pre-specified criteria as it would have been the case in the target trial. However, assessing the quality of trial emulation using pre-specified criteria, we found that our observational analogue closely emulates our target trial, strengthening the confidence one can hold in our main findings. Fifth, given that our study population consists almost exclusively in men, the impact of gender on prognosis could not be assessed. Finally, baseline eGFR, which is an important confounding factor, was missing for 36% of patients. However, values were available for all patients identified as having a CKD (i.e., missing values were observed solely in patients free of CKD). Consequently, baseline CKD stage could be assessed for all patients using observed (i.e., not imputed) baseline eGFR values. Consequently, the new-onset or worsening CKD outcome—relying on CKD stage at baseline—is accurately estimated for all patients included in the trial. In addition, to mitigate the impact of missing values in the estimation of the propensity score, multiple imputations across 100 datasets and several sensitivity analyses using alternative confounding factors were performed.

## Conclusion

In this emulated RCT in 295 patients with leptospirosis, Stage 3 AKI upon admission, and no immediate need for RRT, we did not demonstrate a beneficial effect of early RRT initiation on the risk of death or new-onset or worsening CKD within a year following hospital admission. The timing of RRT initiation in leptospirosis patients with AKI should not depart from that of the general population of critically ill patients and should conform to the general framework of the AKIKI trial and subsequent RCTs.

## Supplementary Information

Supplementary material 1

## Data Availability

Individual data used in preparation of this article cannot be shared without authorization from the French regulatory agency (Commission Nationale de l’Informatique et des Libertés). Programming code used in preparation for this article is publicly available at https://github.com/yjlombardi/eerlepto.
