# Supplementary material for: Immediate or delayed initiation of renal replacement therapy in patients with leptospirosis and acute kidney injury: a target trial emulation
Source: Ann Intensive Care. 2025 May 14;15:65. doi: 10.1186/s13613-025-01477-5 (PMC12078916; doi:10.1186/s13613-025-01477-5)
Supplement: Supplementary file 1 — Supplementary material 1 [file 13613_2025_1477_MOESM1_ESM.docx]

**Supplemental Methods: Steps to emulate the target trial.**

1. **Allocation**: all patients with documented leptospirosis and Stage 3 acute kidney injury (AKI) upon hospital admission were considered in the analysis and categorized according to the allocated treatment strategy.

Patients assigned to the “early” group received renal replacement therapy (RRT) within 48 hours following hospital admission. Patients in the “delayed” group did not receive RRT within 48 hours following hospital admission. Of note, two patients died within 48 hours following hospital admission: one received RRT whereas the second did not: in the main analysis, they were categorized in the “early” and “delayed” group, respectively, consistent with a per protocol analysis.

The protocol for RRT modality remained the same throughout the study period in both centers: RRT was performed using continuous veno-venous hemofiltration (CVVH) at a dose of 30 mL/kg/h, with unfractionated heparin used for anticoagulation when needed.

1. **Outcome adjudication**: patients were considered as having met the primary outcome if a) they died within a year or b) they developed new-onset or worsening CKD.
2. **Imputation for missing data**: multiple imputation using chained equation was used to impute missing baseline values under the “missing at random” assumption. One hundred datasets with imputed values were generated. Outcome variables were included in the dataset used for imputation, as previously described (doi: 10.1016/j.jclinepi.2006.01.009).
3. **Derivation of a propensity score**: a propensity score was derived for each imputed dataset using multivariate logistic regression. The propensity score estimates the probability of treatment allocation (i.e., having RRT initiated within 48 hours), as a function of covariates assessed upon hospital entry. The following covariates were included in the propensity score in the main analysis: age, history of cancer, baseline estimated glomerular filtration rate (eGFR); values upon hospital entry for heart rate, systolic blood pressure, temperature, Glasgow coma scale, arterial partial O2 pressure, blood urea nitrogen, potassium, bicarbonate, sodium, total bilirubin, leukocytes count. These variables were all included in the SAPSII score (except for baseline eGFR) and were selected based on expert opinion because they can be regarded as confounding factors - being, by definition, linked to the initial severity.
4. **Estimation of the treatment effect in each imputed dataset**: in each dataset, the odds ratio (and corresponding 95% confidence interval [CI]) of outcome occurrence according to the treatment strategy was estimated using logistic regression; the extent to which a patient contributed to the analysis was either unweighted (i.e., all patients participated equally to the analysis), or weighted using the inverse of the probability of treatment given by the propensity score (i.e., inverse probability of treatment weighting [IPTW]).
5. **Summary of the treatment effect across datasets**: using Rubin’s rule, the pooled odds ratio and 95% CI (across all datasets) was computed. These pooled values were used to test whether there existed a statistically significant difference of the treatment allocation on the outcome in the main analysis.
6. **Assessment of the balance between groups**: the balance between groups regarding baseline covariates in the unweighted and weighted analyses was assessed by the mean, across all the imputed datasets, of standardized mean differences and variance. Appropriate balance for a given covariate was achieved if the standardized mean difference was <10% and the variance ratio <2. For illustration, summary tables of baseline characteristics according to the treatment strategy are also provided, using data from the first (of 100) imputed dataset, and showing either unweighted or weighted median and interquartile range for continuous variables or number and percentages for binary variables.

**SUPPLEMENTAL TABLES**

| Component | Target trial | Emulation using observational data (“observational analogue”) |
| --- | --- | --- |
| Eligibility | **Inclusion criteria**  Adults with a diagnosis of leptospirosis and Stage 3 AKI upon hospital entry, defined as at least one of the following:  1) Serum creatinine (SCr) > 4 mg/dl (354 µmol/liter)  2) SCr > 3 times the baseline creatinine level  3) Urine output ≤ 100 ml/day for > 12 hours  4) Urine output < 0.3 ml/kg/h for > 24 hours  5) Urine output < 500 ml/day for > 24 hours  **Non-inclusion criteria**  Patients are not included if they have at least one of the following upon hospital entry:  1) Blood urea nitrogen (BUN) > 40 mmol/l  2) Serum potassium > 6 mmol/l  3) Serum bicarbonate < 11 mmol/l  4) Acute pulmonary edema due to fluid overload requiring mechanical ventilation (invasive or non-invasive)  5) Pre-existing chronic kidney disease requiring renal replacement therapy (RRT) | Same as the target trial |
| Treatment strategy | **Early strategy group**  RRT is initiated within 6 hours after randomization and within 48 hours after hospital entry.  **Delayed strategy group**  RRT is initiated if at least one of the following criteria is met:  1) Oliguria or anuria for > 72 hours  2) BUN > 40 mmol/l  3) Serum potassium > 6 mmol/l  4) Serum bicarbonate < 11 mmol/l  5) Acute pulmonary edema due to fluid overload requiring mechanical ventilation (invasive or non-invasive) | Early strategy: same as the target trial  Delayed strategy: RRT is not initiated within 48 hours after hospital entry, and is initiated subsequently only if required (according to local practices) |
| Treatment assignment | Eligible individuals are randomly assigned to one of the two strategies. Both the patient and the treating physician are aware of the assigned treatment strategy (no blinding). | Baseline randomization is emulated by adjusting for baseline confounders |
| Outcomes | **Primary outcome**  Composite criteria, defined as at least one of the following:  - Death within a year following hospital entry  - New-onset or worsening CKD at one year  **Secondary outcomes**  1) Death within a year following hospital entry  2) New-onset or worsening CKD at one year | Same as the target trial |
| Follow-up | Patients are followed-up until death or one year after hospital entry, whichever comes first | Same as the target trial |
| Causal contrast | Intention-to-treat effect | Per protocol effect |
| Analysis plan | Unadjusted logistic regression using treatment allocation as the sole covariate.  Last renal status is carried forward for patients discharged alive without one year follow-up. For instance:   - Considered alive and with new-onset or worsening CKD at one year if dependent to RRT at hospital discharge with no values available at 1, 3 or 12 months, - Considered alive and free of new-onset or worsening CKD at one year if discharged alive with an estimated glomerular filtration rate (eGFR) > 60 ml/min/1.73 m² with no values available at 1, 3 or 12 months, - Considered alive and with new-onset or worsening CKD if with an eGFR at 10 ml/min/1.73 m² at 1 month post-discharge and with no available values at 3 or 12 months. | Weighted (instead of unadjusted) logistic regression is used, using a propensity score estimating the probability of treatment assignment based on baseline confounders  Multiple imputations were used to compute missing values for baseline confounders  Analyses use the same strategy as the target trial to handle missing follow-up information at one year  Mortality and CKD data were assessed by reviewing the electronic health record if the patient returned to the hospital (for a post-hospitalization follow-up consultation or other reason). For patients with no follow-up available in the electronic health records during the year following discharge, follow-up information was obtained by calling the patient or his physician. |

**Table S1.** Specifications for the target trial and its observational analogue.

| **Renal function at baseline according KDIGO CKD nomenclature*** | **New-onset or worsening CKD definition** |
| --- | --- |
| No CKD or Stage I-II CKD | - Need for RRT  - eGFR < 60 ml/min/1.73 m² at last follow-up |
| Stage III CKD | - Need for RRT  - eGFR < 30 ml/min/1.73 m² at last follow-up |
| Stage IV CKD | - Need for RRT  - eGFR < 15 ml/min/1.73 m² at last follow-up |
| Stage V CKD | - Need for RRT |

**Table S2.** New-onset or worsening CKD definition according to renal function at baseline.

*KDIGO: Kidney Diseases Improving Global Outcomes; CKD: chronic kidney disease; RRT: renal replacement therapy; eGFR: estimated glomerular filtration rate.*

*Baseline SCr was collected by assessing medical records prior to hospitalization, and the most recent value obtained at a steady state (i.e., during an outpatient visit) was considered. Baseline eGFR was determined using baseline SCr and the 2021 CKD-EPI equation.*

*Information was missing in 36% of patients. However, no missing values were observed for patients identified in the health records as having a CKD at baseline, thus enabling us to reliably assess baseline CKD stage (I-II; III; IV or V) for all patients, thus enabling us to reliably assess the “new-onset or worsening CKD” outcome for all patients.*

| **Criterion** | **Value** | ***Comments*** |
| --- | --- | --- |
| ***Emulation*** |  |  |
| Comparator emulation | Moderate | *The active comparator (“delayed strategy”) had to be modified for feasibility reasons* |
| Outcome emulation | Good | *All patients had available value for vital or renal status at 1 year* |
| ***Target trial*** |  |  |
| Run-in window | No |  |
| Placebo control | No |  |
| In-hospital start of treatment | Yes |  |
| Dose titration during follow-up | No |  |
| Discontinuance of maintenance therapy at randomization | No |  |
| Delayed effect | No | *Based on the visual evaluation of the survival curves of the AKIKI trial, no violation of the proportionality hazards assumption is anticipated, at least for a 60-day period* |
|  |  |  |
| **Close emulation** | **Yes** | *Inclusion criterion is met: comparator emulation & outcome emulation are at least moderate with at least one classified as good*  *Exclusion criteria b., c. and d. are not met. Exclusion criterion a. does not apply (equivalent to the “linked data available” scenario since in-hospital follow-up data was available for all patients included in our study)* |

**Table S3.** Assessment of the quality of the emulation using criteria adapted from Wang et al (27).

| **Variable** | *N* | **All patients**  **(N = 380)** |
| --- | --- | --- |
| ***Baseline characteristics*** |  |  |
| Male gender | *380* | 356 (94%) |
| Age (years) | *380* | 51 (41, 59) |
| Weight (kg) | *256* | 72 (63, 80) |
| Height (m) | *151* | 1.70 (1.66, 1.76) |
| Occupational hazard at risk for leptospirosis | *308* | 253 (82%) |
| Recent contact with an animal | *292* | 208 (71%) |
| Dyslipidemia | *377* | 48 (13%) |
| Diabetes mellitus | *379* | 68 (18%) |
| Arterial hypertension | *379* | 88 (23%) |
| Chronic alcohol abuse | *350* | 110 (31%) |
| Tobacco abuse | *347* | 158 (46%) |
| Liver cirrhosis | *370* | 8 (2.2%) |
| Chronic respiratory failure | *379* | 28 (7.4%) |
| Heart failure | *378* | 11 (2.9%) |
| Ischemic heart disease | *379* | 15 (4.0%) |
| Immunosuppressive drugs | *379* | 1 (0.3%) |
| Neurological condition | *379* | 18 (4.7%) |
| Cancer | *379* | 15 (4.0%) |
| Chronic kidney disease* | *374* | 25 (6.7%) |
| Chronic hemodialysis | *378* | 1 (0.3%) |
| Baseline creatinine (µmol/l) | *241* | 77.0 (69.0 to 88.0) |
| Baseline eGFR (ml/min/1.73 m²)** | *245* | 101.8 (89.2 to 109.9) |
|  |  |  |
| ***Signs and symptoms upon hospital entry*** |  |  |
| Fever | *378* | 360 (95%) |
| Hypothermia | *378* | 12 (3.2%) |
| Myalgia | *380* | 315 (83%) |
| Arthralgia | *380* | 128 (34%) |
| Jaundice | *379* | 264 (70%) |
| Abdominal pain | *380* | 199 (52%) |
| Nausea/vomiting | *380* | 163 (43%) |
| Hepato- or splenomegaly | *380* | 65 (17%) |
| Headache | *379* | 127 (34%) |
| Dyspnea | *380* | 93 (24%) |
| Cough | *379* | 55 (15%) |
| Livedo | *380* | 134 (35%) |
|  |  |  |
| ***Diagnosis of leptospirosis*** |  |  |
| Positive blood PCR | *355* | 261 (74%) |
| Positive urinary PCR | *153* | 111 (73%) |
| Positive serology | *207* | 184 (89%) |
|  |  |  |
| ***Vital signs upon hospital entry*** |  |  |
| Glasgow coma scale < 15 | *380* | 16 (4.2%) |
| Systolic blood pressure (mmHg) | *368* | 119 (101, 132) |
| Diastolic blood pressure (mmHg) | *368* | 70 (60, 80) |
| Mean blood pressure (mmHg) | *370* | 85 (75, 97) |
| Heart rate (bpm) | *368* | 99 (85, 111) |
| Temperature (°C) | *358* | 37.30 (36.63, 38.30) |
| Respiratory rate (beats per minute) | *99* | 26 (20, 32) |
|  |  |  |
| ***Biology upon hospital entry*** |  |  |
| Arterial pH | *253* | 7.43 (7.38, 7.46) |
| Arterial PO2 (mmHg) | *253* | 85 (73, 99) |
| Arterial PCO2 (mmHg) | *253* | 34 (31, 39) |
| Arterial lactate (mmol/L) | *253* | 1.40 (1.00, 2.20) |
| Arterial base excess (mmol/L) | *246* | -1.9 (-5.0, 1.1) |
| Urea (mmol/L) | *380* | 18 (13, 25) |
| Serum creatinine (μmol/L) | *380* | 368 (230, 534) |
| Blood aspartate aminotransferase (IU/L) | *378* | 121 (70, 214) |
| Blood alanine aminotransferase (IU/L) | *379* | 74 (45, 118) |
| Alkaline phosphatase (IU/l) | *373* | 94 (72, 123) |
| Gamma-GT (IU/L) | *379* | 97 (52, 157) |
| Total bilirubin (μmol/L) | *377* | 104 (36, 205) |
| Lipase (UI/L) | *345* | 54 (31, 130) |
| Leukocytes (G/L) | *379* | 10.2 (7.9, 13.7) |
| Hemoglobin (g/dL) | *380* | 12.50 (11.00, 13.80) |
| Platelets (G/L) | *380* | 48 (29, 82) |
| Prothrombin time (%) | *369* | 84 (73, 93) |
| Partial thromboplastin time (ratio) | *347* | 1.10 (1.00, 1.21) |
| Fibrinogen (g/L) | *280* | 8.00 (7.08, 9.13) |
| Factor V activity (%) | *55* | 123 (98, 150) |
| Lactate dehydrogenase (UI/L) | *230* | 649 (487, 910) |
| Creatine phosphokinase (IU/L) | *356* | 1,869 (676, 4,740) |
| Troponin (µg/L) | *283* | 14 (2, 39) |
| Brain natriuretic peptide (pg/ml) | *14* | 204 (68, 633) |
| Uric acid (µmol/L) | *225* | 450 (372, 563) |
| C-reactive protein (mg/L) | *315* | 201 (128, 296) |
| Procalcitonin (µg/L) | *167* | 5 (2, 11) |
| Sodium (mmol/L) | *379* | 133 (130, 136) |
| Potassium (mmol/L) | *379* | 3.50 (3.20, 3.90) |
| Bicarbonate (mmol/L) | *378* | 22.0 (19.0, 24.0) |
| Chloride (mmol/L) | *377* | 93 (89, 97) |
| Phosphate (mmol/L) | *364* | 1.28 (1.02, 1.61) |
| Calcium (mmol/L) | *366* | 2.11 (1.99, 2.22) |
| Magnesium (mmol/L) | *278* | 0.92 (0.78, 1.07) |
| Leukocyturia | *256* | 177 (69%) |
| Urinary chemistry performed | *341* | 205 (60%) |
|  |  |  |
| ***Treatments during hospitalization*** |  |  |
| Antibiotherapy | *380* | 375 (99%) |
| Time to antibiotherapy initiation (days) | *364* | 5 (3, 6) |
| Antibiotherapy duration (days) | *374* | 7 (7, 7) |
| Transfusion of red blood cells or platelets | *355* | 114 (32%) |
| ICU admission | *380* | 271 (71%) |
| Length of ICU stay (days) | *271* | 4 (0, 6) |
| Vasoactive drug | *380* | 84 (22%) |
| Invasive ventilation | *380* | 69 (18%) |
| RRT initiation | *380* | 149 (39%) |
| Time to RRT initiation (days) | *148* | 1.00 (0.00, 2.00) |
| RRT duration (days) | *147* | 4 (3, 11) |
| Type of RRT | *149* |  |
| CVVH only |  | 101 (68%) |
| CVVH then HD |  | 31 (21%) |
| HD only |  | 15 (10%) |
| IHD then CVVH |  | 2 (1.3%) |
| Urea at RRT initiation (mmol/L) | *145* | 29 (21, 39) |
| Creatinine at RRT initiation (µmol/L) | *146* | 613 (433, 783) |
| Potassium at RRT initiation (mmol/L) | *146* | 3.90 (3.55, 4.40) |
| Bicarbonate at RRT initiation (mmol/L) | *145* | 18.0 (16.0, 21.0) |
| Urea at RRT end (mmol/L) | *145* | 13 (8, 20) |
| Creatinine at RRT end (mmol/L) | *146* | 224 (146, 391) |
| Length of hospital stay (days) | *380* | 9 (6, 14) |
| Urea at discharge (mmol/L) | *369* | 10 (7, 16) |
| Creatinine at discharge (µmol/L) | *370* | 123 (94, 213) |
|  |  |  |
| ***Events during hospitalization*** |  |  |
| Type 2 ACS and/or myocarditis and/or pericarditis | *380* | 52 (14%) |
| Hemorrhagic syndrome | *380* | 44 (12%) |
| Cardiac arrhythmia | *380* | 94 (25%) |
| Oligo-anuria | *374* | 218 (58%) |
| Intra-alveolar hemorrhage | *380* | 41 (11%) |
| Hemoptysis | *380* | 86 (23%) |
| ARDS | *380* | 44 (12%) |
| Gastrointestinal bleeding | *380* | 33 (8.7%) |
| Meningitis and/or encephalitis | *380* | 49 (13%) |
| Intracranial hemorrhage | *380* | 4 (1.1%) |
| In-hospital death | *380* | 16 (4.2%) |
| Time to in-hospital death (days) | *16* | 3.50 (2.75, 6.25) |
|  |  |  |
| **Follow-up after hospital discharge** |  |  |
| Death between discharge and month 12 | *364* | 2 (0.5%) |
| Dependency to RRT at month 12 | *364* | 4 (1.1%) |
| Urea at month 1 (mmol/L) | *120* | 6 (5, 9) |
| Creatinine at month 1 (µmol/L) | *175* | 100 (84, 129) |
| Potassium at month 1 (mmol/L) | *106* | 4.05 (3.80, 4.40) |
| Calcium at month 1 (mmol/L) | *52* | 2.36 (2.27, 2.42) |
| Hemoglobin at month 1 (mmol/L) | *152* | 10.80 (9.10, 12.13) |
| Urea at month 3 (mmol/L) | *42* | 6.0 (4.5, 8.5) |
| Creatinine at month 3 (µmol/L) | *61* | 95 (79, 131) |
| Potassium at month 3 (mmol/L) | *44* | 4.00 (3.70, 4.50) |
| Calcium at month 3 (mmol/L) | *24* | 2.37 (2.33, 2.44) |
| Hemoglobin at month 3 (mmol/L) | *41* | 12.70 (10.90, 13.90) |
| Urea at month 12 (mmol/L) | *65* | 5.1 (4.1, 6.2) |
| Creatinine at month 12 (µmol/L) | *103* | 88 (76, 106) |
| Potassium at month 12 (mmol/L) | *63* | 3.90 (3.70, 4.33) |
| Calcium at month 12 (mmol/L) | *35* | 2.31 (2.24, 2.38) |
| Hemoglobin at month 12 (mmol/L) | *63* | 14.40 (12.80, 15.65) |

**Table S4.** Full characteristics and outcomes for patients included in the cohort study.
*ARDS: acute respiratory distress; bpm: beats per minute; CVVH: continuous veno-venous hemofiltration; eGFR: estimated glomerular filtration rate; HD: hemodialysis; ICU: intensive care unit; IHD: intermittent hemodialysis; PCO2: partial pressure of carbon dioxide; PCR: polymerase chain reaction; PO2: partial pressure of oxygen; RRT: renal replacement therapy*

**Chronic kidney disease explicitly stated as a medical history in the medical report*

***For patients with a baseline serum creatinine value available, baseline estimated glomerular filtration rate (eGFR) was derived using the race-free 2021 Chronic Kidney Disease Epidemiology Collaboration (CKD-EPI) equation; for patients with no baseline serum creatinine value available but with a baseline eGFR value explicitly stated in the medical report, this value was considered.*

| **Variable** | *N* | **All patients**  **(N = 295)** | *N* | **Delayed strategy**  **(N = 213)** | *N* | **Early strategy**  **(N = 82)** | **SMD**  **(%)** |
| --- | --- | --- | --- | --- | --- | --- | --- |
| ***Baseline characteristics*** |  |  |  |  |  |  |  |
| Male gender | *295* | 275 (93%) | *213* | 196 (92%) | *82* | 79 (96%) | **19%** |
| Age (years) | *295* | 52 (41, 59) | *213* | 51 (42, 59) | *82* | 52 (39, 60) | **11%** |
| Weight (kg) | *207* | 70 (62, 78) | *141* | 72 (63, 80) | *66* | 70 (61, 77) | **26%** |
| Height (m) | *120* | 1.70 (1.66, 1.76) | *71* | 1.72 (1.67, 1.76) | *49* | 1.69 (1.64, 1.76) | **29%** |
| Occupational hazard at risk for leptospirosis | *244* | 199 (82%) | *179* | 150 (84%) | *65* | 49 (75%) | **21%** |
| Recent contact with an animal | *230* | 166 (72%) | *167* | 117 (70%) | *63* | 49 (78%) | **18%** |
| Dyslipidemia | *293* | 36 (12%) | *211* | 31 (15%) | *82* | 5 (6.1%) | **28%** |
| Diabetes mellitus | *295* | 53 (18%) | *213* | 43 (20%) | *82* | 10 (12%) | **22%** |
| Arterial hypertension | *295* | 66 (22%) | *213* | 48 (23%) | *82* | 18 (22%) | 1% |
| Chronic alcohol abuse | *274* | 89 (32%) | *198* | 59 (30%) | *76* | 30 (39%) | **20%** |
| Tobacco abuse | *272* | 128 (47%) | *197* | 89 (45%) | *75* | 39 (52%) | **14%** |
| Liver cirrhosis | *290* | 6 (2.1%) | *210* | 3 (1.4%) | *80* | 3 (3.8%) | **15%** |
| Chronic respiratory failure | *295* | 21 (7.1%) | *213* | 16 (7.5%) | *82* | 5 (6.1%) | 6% |
| Heart failure | *294* | 8 (2.7%) | *212* | 6 (2.8%) | *82* | 2 (2.4%) | 2% |
| Ischemic heart disease | *295* | 10 (3.4%) | *213* | 8 (3.8%) | *82* | 2 (2.4%) | 8% |
| Immunosuppressive drugs | *295* | 1 (0.3%) | *213* | 1 (0.5%) | *82* | 0 (0%) | **10%** |
| Neurological condition | *295* | 13 (4.4%) | *213* | 8 (3.8%) | *82* | 5 (6.1%) | **11%** |
| Cancer | *295* | 11 (3.7%) | *213* | 9 (4.2%) | *82* | 2 (2.4%) | **10%** |
| Baseline creatinine (µmol/l)* | *187* | 77.0 (67.0 to 87.0) | *141* | 75.0 (67.0 to 87.0) | *46* | 79.0 (69.3 to 88.0) | 9% |
| Baseline eGFR (ml/min/1.73 m²)* | *188* | 102.9 (90.2 to 111.3) | *142* | 103.2 (90.8 to 109.6) | *46* | 102.4 (86.9 to 116.5) | **12%** |
| Status regarding chronic kidney disease (CKD)** | *295* |  | *213* |  | *82* |  | 7% |
| No CKD |  | 277 (93.9%) |  | 199 (93.4%) |  | 78 (95.1%) |  |
| CKD |  | 18 (6.1%) |  | 14 (6.6%) |  | 4 (4.9%) |  |
| Stage I-II*** |  | 7 (2.4%) |  | 5 (2.3%) |  | 2 (2.4%) |  |
| Stage III*** |  | 8 (2.7%) |  | 6 (2.8%) |  | 2 (2.4%) |  |
| Stage IV-V*** |  | 3 (0.1%) |  | 3 (1.4%) |  | 0 (0.0%) |  |
|  |  |  |  |  |  |  |  |
| ***Signs and symptoms upon hospital entry*** |  |  |  |  |  |  |  |
| Fever | *293* | 280 (96%) | *211* | 201 (95%) | *82* | 79 (96%) | 5% |
| Hypothermia | *294* | 10 (3.4%) | *212* | 1 (0.5%) | *82* | 9 (11%) | **46%** |
| Myalgia | *295* | 251 (85%) | *213* | 186 (87%) | *82* | 65 (79%) | **22%** |
| Arthralgia | *295* | 104 (35%) | *213* | 87 (41%) | *82* | 17 (21%) | **45%** |
| Jaundice | *295* | 225 (76%) | *213* | 156 (73%) | *82* | 69 (84%) | **27%** |
| Abdominal pain | *295* | 152 (52%) | *213* | 104 (49%) | *82* | 48 (59%) | **20%** |
| Nausea/vomiting | *295* | 129 (44%) | *213* | 90 (42%) | *82* | 39 (48%) | **11%** |
| Hepato- or splenomegaly | *295* | 55 (19%) | *213* | 36 (17%) | *82* | 19 (23%) | **16%** |
| Headache | *294* | 97 (33%) | *213* | 75 (35%) | *81* | 22 (27%) | **17%** |
| Dyspnea | *295* | 76 (26%) | *213* | 43 (20%) | *82* | 33 (40%) | **45%** |
| Cough | *294* | 43 (15%) | *212* | 34 (16%) | *82* | 9 (11%) | **15%** |
| Livedo | *295* | 103 (35%) | *213* | 55 (26%) | *82* | 48 (59%) | **70%** |
|  |  |  |  |  |  |  |  |
| ***Diagnosis of leptospirosis*** |  |  |  |  |  |  |  |
| Positive blood PCR | *278* | 210 (76%) | *200* | 143 (72%) | *78* | 67 (86%) | **36%** |
| Positive urinary PCR | *121* | 91 (75%) | *77* | 55 (71%) | *44* | 36 (82%) | **25%** |
| Positive serology | *150* | 134 (89%) | *122* | 109 (89%) | *28* | 25 (89%) | 0% |
|  |  |  |  |  |  |  |  |
| ***Vital signs upon hospital entry*** |  |  |  |  |  |  |  |
| Glasgow coma scale < 15 | *295* | 11 (3.7%) | *213* | 5 (2.3%) | *82* | 6 (7.3%) | **23%** |
| Systolic blood pressure (mmHg) | *289* | 119 (101, 131) | *209* | 120 (107, 132) | *80* | 110 (90, 130) | **46%** |
| Diastolic blood pressure (mmHg) | *289* | 70 (60, 80) | *209* | 71 (60, 80) | *80* | 65 (52, 79) | **42%** |
| Mean blood pressure (mmHg) | *290* | 86 (76, 98) | *209* | 88 (77, 98) | *81* | 82 (63, 93) | **49%** |
| Heart rate (bpm) | *288* | 100 (85, 111) | *206* | 96 (85, 110) | *82* | 107 (94, 120) | **39%** |
| Temperature (°C) | *280* | 37.30 (36.60, 38.20) | *200* | 37.30 (36.70, 38.30) | *80* | 37.20 (36.45, 38.10) | **23%** |
| Respiratory rate (beats per minute) | *83* | 26 (20, 30) | *58* | 26 (20, 30) | *25* | 26 (22, 34) | **10%** |
|  |  |  |  |  |  |  |  |
| ***Biology upon hospital entry*** |  |  |  |  |  |  |  |
| Arterial pH | *204* | 7.43 (7.39, 7.46) | *126* | 7.44 (7.41, 7.47) | *78* | 7.40 (7.34, 7.44) | **77%** |
| Arterial PO2 (mmHg) | *204* | 85 (74, 98) | *126* | 83 (71, 96) | *78* | 89 (78, 101) | **31%** |
| Arterial PCO2 (mmHg) | *204* | 34 (31, 39) | *126* | 33 (31, 38) | *78* | 35 (31, 41) | **22%** |
| Arterial lactate (mmol/L) | *203* | 1.40 (1.10, 2.25) | *124* | 1.30 (1.00, 1.93) | *79* | 1.80 (1.20, 2.70) | **50%** |
| Arterial base excess (mmol/L) | *200* | -2.0 (-5.0, 1.4) | *124* | -1.0 (-3.3, 2.0) | *76* | -4.0 (-7.0, -1.0) | **73%** |
| Urea (mmol/L) | *295* | 20 (15, 26) | *213* | 19 (14, 25) | *82* | 23 (17, 30) | **52%** |
| Serum creatinine (μmol/L) | *295* | 406 (301, 550) | *213* | 378 (287, 512) | *82* | 509 (370, 627) | **53%** |
| Blood aspartate aminotransferase (IU/L) | *293* | 129 (78, 231) | *211* | 121 (70, 217) | *82* | 167 (90, 242) | **16%** |
| Blood alanine aminotransferase (IU/L) | *294* | 76 (46, 116) | *212* | 76 (46, 114) | *82* | 78 (47, 119) | **13%** |
| Alkaline phosphatase (IU/l) | *291* | 93 (73, 120) | *209* | 96 (75, 125) | *82* | 90 (71, 110) | **31%** |
| Gamma-GT (IU/L) | *294* | 97 (52, 151) | *212* | 103 (57, 160) | *82* | 76 (44, 136) | **20%** |
| Total bilirubin (μmol/L) | *293* | 126 (51, 214) | *211* | 114 (44, 205) | *82* | 157 (77, 252) | **35%** |
| Lipase (UI/L) | *270* | 54 (30, 124) | *195* | 51 (28, 109) | *75* | 67 (40, 186) | **44%** |
| Leukocytes (G/L) | *294* | 10.5 (8.2, 14.1) | *212* | 10.3 (8.2, 13.3) | *82* | 11.4 (8.6, 14.6) | **28%** |
| Hemoglobin (g/dL) | *295* | 12.50 (10.85, 13.75) | *213* | 12.80 (11.30, 13.90) | *82* | 11.30 (9.28, 13.18) | **31%** |
| Platelets (G/L) | *295* | 44 (26, 70) | *213* | 45 (30, 75) | *82* | 35 (19, 59) | **42%** |
| Prothrombin time (%) | *288* | 84 (75, 93) | *206* | 85 (76, 94) | *82* | 81 (70, 89) | **38%** |
| Partial thromboplastin time (ratio) | *270* | 1.10 (1.00, 1.21) | *197* | 1.10 (1.00, 1.20) | *73* | 1.10 (1.00, 1.22) | **11%** |
| Fibrinogen (g/L) | *217* | 8.10 (7.20, 9.30) | *167* | 8.10 (7.30, 9.30) | *50* | 7.56 (6.81, 9.17) | **20%** |
| Factor V activity (%) | *41* | 128 (102, 150) | *14* | 129 (119, 157) | *27* | 121 (97, 150) | **28%** |
| Lactate dehydrogenase (UI/L) | *194* | 665 (504, 951) | *139* | 658 (498, 865) | *55* | 768 (522, 1,093) | **28%** |
| Creatine phosphokinase (IU/L) | *282* | 2,226 (819, 5,567) | *206* | 2,046 (804, 5,459) | *76* | 2,477 (895, 5,586) | **10%** |
| Troponin (µg/L) | *227* | 15 (2, 43) | *150* | 14 (4, 34) | *77* | 23 (2, 64) | **19%** |
| Brain natriuretic peptide (pg/ml) | *9* | *226 (72, 673)* | *6* | 449.5 (82, 1625.5) | *3* | 182 (127, 204.5) | **110%** |
| Uric acid (µmol/L) | *183* | 455 (373, 562) | *152* | 449 (377, 542) | *31* | 519 (316, 624) | **14%** |
| C-reactive protein (mg/L) | *246* | 210 (132, 299) | *196* | 200 (128, 302) | *50* | 239 (156, 296) | **12%** |
| Procalcitonin (µg/L) | *135* | 6 (2, 11) | *97* | 5 (2, 9) | *38* | 6 (3, 28) | **46%** |
| Sodium (mmol/L) | *295* | 133 (130, 136) | *213* | 133 (130, 136) | *82* | 132 (130, 135) | **10%** |
| Potassium (mmol/L) | *294* | 3.56 (3.20, 3.90) | *212* | 3.50 (3.15, 3.82) | *82* | 3.70 (3.30, 4.08) | **33%** |
| Bicarbonate (mmol/L) | *294* | 22.0 (19.0, 24.0) | *212* | 22.0 (20.0, 24.0) | *82* | 20.0 (17.0, 22.0) | **62%** |
| Chloride (mmol/L) | *294* | 93 (89, 96) | *212* | 93 (89, 96) | *82* | 94 (88, 98) | **15%** |
| Phosphate (mmol/L) | *288* | 1.33 (1.06, 1.63) | *206* | 1.30 (1.02, 1.54) | *82* | 1.45 (1.11, 1.79) | **42%** |
| Calcium (mmol/L) | *290* | 2.11 (1.99, 2.22) | *208* | 2.14 (2.05, 2.23) | *82* | 2.01 (1.87, 2.12) | **62%** |
| Magnesium (mmol/L) | *225* | 0.93 (0.79, 1.06) | *147* | 0.92 (0.79, 1.05) | *78* | 0.95 (0.78, 1.07) | **14%** |
| Leukocyturia | *207* | 150 (72%) | *146* | 100 (68%) | *61* | 50 (82%) | **32%** |
| Urinary chemistry performed | *263* | 171 (65%) | *196* | 118 (60%) | *67* | 53 (79%) | **42%** |
|  |  |  |  |  |  |  |  |
| ***Treatments during hospitalization*** |  |  |  |  |  |  |  |
| Antibiotherapy | *295* | 291 (99%) | *213* | 210 (99%) | *82* | 81 (99%) | 2% |
| Time to antibiotherapy initiation (days) | *282* | 5 (3, 6) | *204* | 5 (3, 6) | *78* | 5 (4, 7) | **13%** |
| Antibiotherapy duration (days) | *291* | 7 (7, 7) | *210* | 7 (7, 7) | *81* | 7 (7, 7) | **30%** |
| Transfusion of red blood cells or platelets | *280* | 96 (34%) | *204* | 48 (24%) | *76* | 48 (63%) | **87%** |
| ICU admission | *295* | 223 (76%) | *213* | 141 (66%) | *82* | 82 (100%) | **100%** |
| Length of ICU stay (days) | *295* | 4 (2, 7) | *213* | 3 (0, 5) | *82* | 7 (5, 11) | **82%** |
| Vasoactive drug | *295* | 67 (23%) | *213* | 30 (14%) | *82* | 37 (45%) | **72%** |
| Invasive ventilation | *295* | 58 (20%) | *213* | 22 (10%) | *82* | 36 (44%) | **82%** |
| RRT initiation | *295* | 135 (46%) | *213* | 53 (25%) | *82* | 82 (100%) | **250%** |
| Time to RRT initiation (days) | *135* | *2.0 (1.0, 3.0)* | *53* | 4.0 (3.0, 5.0) | *82* | 1.0 (1.0, 2.0) | **280%** |
| RRT duration (days) | *134* | 4 (2, 10) | *52* | 3 (1, 9) | *82* | 4 (3, 12) | **34%** |
| Type of RRT | *135* |  | *53* |  | *82* |  | **90%** |
| CVVH only |  | 93 (69%) |  | 31 (58%) |  | 62 (76%) |  |
| CVVH then HD |  | 27 (20%) |  | 7 (13%) |  | 20 (24%) |  |
| HD only |  | 13 (9.6%) |  | 13 (25%) |  | 0 (0%) |  |
| IHD then CVVH |  | 2 (1.5%) |  | 2 (3.8%) |  | 0 (0%) |  |
| Urea at RRT initiation (mmol/L) | *132* | 29 (21, 37) | *52* | 36 (28, 51) | *80* | 24 (19, 32) | **120%** |
| Creatinine at RRT initiation (µmol/L) | *133* | 601 (430, 774) | *52* | 704 (552, 877) | *81* | 523 (383, 658) | **74%** |
| Potassium at RRT initiation (mmol/L) | *133* | 3.90 (3.50, 4.34) | *52* | 4.21 (3.78, 4.64) | *81* | 3.70 (3.30, 4.00) | **73%** |
| Bicarbonate at RRT initiation (mmol/L) | *132* | 18.0 (16.0, 21.0) | *52* | 17.0 (14.8, 19.0) | *80* | 19.5 (17.0, 22.0) | **64%** |
| Urea at RRT end (mmol/L) | *132* | 12 (8, 20) | *51* | 16 (10, 22) | *81* | 11 (7, 16) | **59%** |
| Creatinine at RRT end (mmol/L) | *133* | 220 (146, 383) | *51* | 318 (193, 519) | *82* | 177 (126, 291) | **56%** |
| Length of hospital stay (days) | *295* | 9 (7, 15) | *213* | 8 (7, 12) | *82* | 14 (10, 22) | **54%** |
| Urea at discharge (mmol/L) | *287* | 11 (8, 19) | *207* | 10 (8, 18) | *80* | 13 (8, 21) | 7% |
| Creatinine at discharge (µmol/L) | *287* | 144 (99, 255) | *207* | 140 (98, 225) | *80* | 178 (103, 319) | **22%** |
| eGFR at discharge (ml/min/1.73 m²) | *287* | 51 (25, 77) | *207* | 52 (29, 78) | *80* | 39 (20, 74) | **12%** |
|  |  |  |  |  |  |  |  |
| ***Events during hospitalization*** |  |  |  |  |  |  |  |
| Type 2 ACS and/or myocarditis and/or pericarditis | *295* | 43 (15%) | *213* | 24 (11%) | *82* | 19 (23%) | **32%** |
| Hemorrhagic syndrome | *295* | 35 (12%) | *213* | 20 (9.4%) | *82* | 15 (18%) | **26%** |
| Cardiac arrhythmia | *295* | 77 (26%) | *213* | 47 (22%) | *82* | 30 (37%) | **32%** |
| Oligo-anuria | *291* | 189 (65%) | *210* | 118 (56%) | *81* | 71 (88%) | **75%** |
| Intra-alveolar hemorrhage | *295* | 30 (10%) | *213* | 17 (8.0%) | *82* | 13 (16%) | **24%** |
| Hemoptysis | *295* | 69 (23%) | *213* | 45 (21%) | *82* | 24 (29%) | **19%** |
| ARDS | *295* | 36 (12%) | *213* | 15 (7.0%) | *82* | 21 (26%) | **52%** |
| Gastrointestinal bleeding | *295* | 26 (8.8%) | *213* | 13 (6.1%) | *82* | 13 (16%) | **32%** |
| Meningitis and/or encephalitis | *295* | 36 (12%) | *213* | 17 (8.0%) | *82* | 19 (23%) | **43%** |
| Intracranial hemorrhage | *295* | 4 (1.4%) | *213* | 2 (0.9%) | *82* | 2 (2.4%) | **12%** |
| In-hospital death | *295* | 12 (4.1%) | *213* | 8 (3.8%) | *82* | 4 (4.9%) | 6% |
| Time to in-hospital death (days) | *12* | 3.50 (3.00, 6.50) | *8* | 3.50 (3.00, 5.25) | *4* | 6.0 (2.75, 9.25) | **62%** |
|  |  |  |  |  |  |  |  |
| **Follow-up after hospital discharge** |  |  |  |  |  |  |  |
| Death between discharge and month 12 | *283* | 2 (0.7%) | *205* | 1 (0.5%) | *78* | 1 (1.3%) | 9% |
| Potassium at month 1 (mmol/L) | *92* | 4.08 (3.80, 4.40) | *61* | 4.03 (3.80, 4.30) | *31* | 4.20 (3.80, 4.45) | 2% |
| Calcium at month 1 (mmol/L) | *46* | 2.36 (2.27, 2.42) | *30* | 2.35 (2.27, 2.41) | *16* | 2.36 (2.29, 2.43) | **10%** |
| Hemoglobin at month 1 (mmol/L) | *130* | 10.55 (9.00, 11.98) | *85* | 11.00 (9.40, 12.10) | *45* | 9.40 (8.60, 11.40) | **53%** |
| Potassium at month 3 (mmol/L) | *33* | 4.00 (3.70, 4.60) | *18* | 4.25 (3.75, 4.68) | *15* | 3.90 (3.75, 4.15) | **33%** |
| Calcium at month 3 (mmol/L) | *18* | 2.37 (2.34, 2.45) | *9* | 2.38 (2.35, 2.52) | *9* | 2.36 (2.34, 2.41) | **12%** |
| Hemoglobin at month 3 (mmol/L) | *31* | 12.70 (10.95, 13.80) | *17* | 13.50 (12.50, 15.00) | *14* | 11.80 (10.60, 12.63) | **73%** |
| Potassium at month 12 (mmol/L) | *51* | 3.90 (3.60, 4.38) | *37* | 3.90 (3.60, 4.40) | *14* | 3.85 (3.60, 4.28) | **14%** |
| Calcium at month 12 (mmol/L) | *31* | 2.31 (2.24, 2.36) | *25* | 2.30 (2.23, 2.35) | *6* | 2.33 (2.30, 2.46) | **37%** |
| Hemoglobin at month 12 (mmol/L) | *52* | 14.00 (12.60, 15.33) | *38* | 14.40 (12.85, 15.38) | *14* | 11.80 (11.28, 14.83) | **74%** |
|  |  |  |  |  |  |  |  |
| **Kidney function after hospital discharge** |  |  |  |  |  |  |  |
| Dependency to RRT at month 12 | *283* | 4 (1.4%) | *205* | 3 (1.5%) | *78* | 1 (1.3%) | 2% |
| eGFR at month 1 (ml/min/1.73 m²) | *148* | 78 (53, 96) | *100* | 82 (65, 98) | *48* | 58 (46, 90) | **50%** |
| eGFR at month 3 (ml/min/1.73 m²) | *48* | 84 (55, 101) | *26* | 84 (58, 95) | *22* | 84 (54, 103) | 5% |
| eGFR at month 12 (ml/min/1.73 m²) | *90* | 90 (73, 106) | *67* | 90 (74, 105) | *23* | 89 (70, 105) | 1% |
| Last known value for eGFR at month 12 (ml/min/1.73 m²) | *287* | 85 (65, 103) | *207* | 85 (69, 102) | *80* | 80 (53, 104) | **14%** |
| Creatinine at month 1 (µmol/L) | *142* | 101 (83, 131) | *94* | 93 (81, 116) | *48* | 118 (92, 163) | **31%** |
| Creatinine at month 3 (µmol/L) | *47* | 94 (80, 130) | *25* | 97 (80, 129) | *22* | 92 (80, 130) | 2% |
| Creatinine at month 12 (µmol/L) | *87* | 87 (74, 106) | *65* | 85 (74, 105) | *22* | 90 (75, 107) | 5% |
| Urea at month 1 (mmol/L) | *102* | 6 (5, 8) | *67* | 6 (5, 8) | *35* | 6 (5, 11) | **31%** |
| Urea at month 3 (mmol/L) | *32* | 6.0 (4.8, 8.9) | *19* | 5.9 (4.8, 8.7) | *13* | 6.1 (4.9, 9.5) | **16%** |
| Urea at month 12 (mmol/L) | *55* | 5.0 (4.0, 6.2) | *41* | 5.1 (4.1, 6.2) | *14* | 4.7 (3.8, 5.7) | **14%** |

**Table S5.** Full characteristics and outcomes of patients included in the emulation study, according to the treatment strategy.

*ACS: acute coronary syndrome; ARDS: acute respiratory distress; bpm: beats per minute; CVVH: continuous veno-venous hemofiltration; eGFR: estimated glomerular filtration rate; HD: hemodialysis; ICU: intensive care unit; IHD: intermittent hemodialysis; PCO2: partial pressure of carbon dioxide; PCR: polymerase chain reaction; PO2: partial pressure of oxygen; RRT: renal replacement therapy; SMD: absolute standardized mean difference.*

**For patients with a baseline serum creatinine value available, baseline eGFR was derived using the race-free 2021 Chronic Kidney Disease Epidemiology Collaboration (CKD-EPI) equation; for patients with no baseline serum creatinine value available but with a baseline eGFR value explicitly stated in the medical report, this value was considered.*

***Status regarding chronic kidney disease at baseline was assessable for all patients using data collected (i.e., not imputed) and was determined as:*

- *No CKD: no mention of history of CKD, either in the medical report of the index stay or in previous medical reports,*
- *CKD: mention of history of CKD in a medical report*

**** CKD stage at baseline was assessable for all patients using data collected (i.e., not imputed) since all patients with a mention of history of CKD had available data for baseline eGFR (i.e., missing eGFR values were solely observed in patients free of CKD). CKD stage was determined as:*

- *No CKD: no mention of history of CKD, either in the medical report of the index stay or in previous medical reports,*
- *Stage I or II: mention of history of CKD in a medical report and baseline eGFR > 60 ml/min/1.73 m²*
- *Stage III: mention of history of CKD in a medical report and baseline eGFR [60-30[ ml/min/1.73 m²*
- *Stage IV-V: mention of history of CKD in a medical report and baseline eGFR ≤30 ml/min/1.73 m²*

| **Variable** | **Before weighting** | |  | **After weighting** | |
| --- | --- | --- | --- | --- | --- |
|  | **Odds ratio**  **(95% CI)** | **p-value** |  | **Odds ratio**  **(95% CI)** | **p-value** |
|  |  |  |  |  |  |
| ***Main analysis*** | 2.78 (1.53 to 5.04) | **<0.001** |  | 2.08 (1.01 to 4.26) | **0.046** |
|  |  |  |  |  |  |
| ***To assess the impact of covariates used for weighting*** |  |  |  |  |  |
| Initial severity assessed using SAPS II (i.e., SAPS II is the sole variable included) |  |  |  | 1.34 (0.65 to 2.77) | 0.430 |
| Initial severity assessed using SOFA (i.e., SOFA is the sole variable included) |  |  |  | 1.69 (0.77 to 3.74) | 0.193 |
| Initial severity assessed using SOFA and SAPS II |  |  |  | 1.25 (0.53 to 2.96) | 0.606 |
| Initial respiratory severity assessed using “Dyspnea” instead of “PO2” |  |  |  | 1.85 (0.86 to 3.95) | 0.114 |
| Initial respiratory severity assessed using “Intra-alveolar hemorrhage” instead of “PO2” |  |  |  | 2.13 (1.03 to 4.39) | **0.041** |
| Initial neurological severity assessed using “Meningo-encephalitis” instead of “GCS” |  |  |  | 2.38 (1.13 to 5.02) | **0.022** |
| Initial neurological severity assessed using “Intracranial hemorrhage” instead of “GCS” |  |  |  | 2.10 (1.02 to 4.33) | **0.045** |
| Initial renal severity assessed using “Baseline creatinine” instead of “Baseline eGFR” |  |  |  | 2.11 (1.03 to 4.34) | **0.041** |
| Initial renal severity assessed using “Day 0 creatinine” instead of “Day 0 BUN” |  |  |  | 2.44 (1.16 to 5.13) | **0.019** |
| Additional covariate: CPK (associated with an increase in risk for RRT) |  |  |  | 2.06 (0.99 to 4.28) | 0.052 |
| Additional covariate: SAPS II |  |  |  | 1.59 (0.64 to 3.92) | 0.317 |
| Additional covariate: SOFA |  |  |  | 1.89 (0.82 to 4.32) | 0.133 |
| Additional covariates: SAPS II and SOFA |  |  |  | 1.50 (0.57 to 3.94) | 0.410 |
|  |  |  |  |  |  |
| ***To assess the impact of functional forms used for weighting*** |  |  |  |  |  |
| eGFR is transformed to sqrt(eGFR) |  |  |  | 2.09 (1.01 to 4.31) | **0.047** |
| eGFR is transformed to log(eGFR) |  |  |  | 2.11 (1.01 to 4.39) | **0.046** |
|  |  |  |  |  |  |
| ***To assess the impact of the method used for weighting*** |  |  |  |  |  |
| “Gradient boosting model” is used instead of “glm” |  |  |  | 2.29 (1.21 to 4.35) | **0.011** |
| “Covariate balancing” is used instead of “glm” |  |  |  | 2.13 (1.07 to 4.22) | **0.031** |
| “Bayesian additive regression trees” is used instead of “glm” |  |  |  | 2.31 (1.23 to 4.36) | **0.010** |
|  |  |  |  |  |  |
| ***To assess the impact of the exclusion strategy*** |  |  |  |  |  |
| A cutoff of bicarbonate “<8” is used instead of “<11” (n = 297) | 2.83 (1.56 to 5.12) | **<0.001** |  | 2.09 (1.02 to 4.27) | **0.044** |
| A cutoff of potassium “>5.5” is used instead of “>6” (n = 294) | 2.76 (1.52 to 5.03) | **<0.001** |  | 2.11 (1.01 to 4.41) | **0.046** |
| A cutoff of BUN “>30” is used instead of “>40” (n = 250) | 2.57 (1.24 to 5.30) | **0.011** |  | 2.62 (1.13 to 6.05) | **0.025** |
| Patients with “intra-alveolar hemorrhage” are excluded (n = 265) | 2.93 (1.52 to 5.64) | **0.001** |  | 2.59 (1.09 to 6.12) | **0.030** |
| Patients with “PCO2 > 50 mmHg” are excluded (n = 290) | 2.59 (1.41 to 4.77) | **0.002** |  | 1.84 (0.86 to 3.92) | 0.114 |
|  |  |  |  |  |  |
| ***To assess the impact of including the two patients who died within 48 hours*** |  |  |  |  |  |
| Patients who died within 48 hours are excluded (n = 293) | 2.76 (1.5 to 5.06) | **0.001** |  | 2.12 (1.02 to 4.42) | **0.044** |

**Table S6.** Treatment effect in the unweighted and weighted study populations in different sensitivity analyses.

*BUN: blood urea nitrogen: CI: confidence interval; CPK: creatinine phosphokinase; eGFR: estimated glomerular filtration rate: GCS: Glasgow coma scale; PCO2: partial pressure of carbon dioxide; PO2: partial pressure of oxygen; RRT: renal replacement therapy; SAPS II: Simplified Acute Physiology Score; SOFA: Sequential Organ Failure Assessment*

***Rationale for sensitivity analyses:*** *each analysis assesses whether changing a key parameter from the main statistical analysis has an effect on the study conclusion.*

***Interpretation:***

1. *All analyses show a point estimate for the odds-ratio > 1, and all analyses have a lower bound for the confidence intervals either containing 1 or greater than 1 🡪 this confirms the robustness of the conclusion of the main analysis (i.e., early RRT is not associated with better outcomes)*
2. *All analyses do not show an OR significantly > 1 🡪 thus, one cannot confirm that early RRT is associated with worst outcomes with certainty that early RRT*

| **Variable** | **Before weighting** | |  | **After weighting** | |
| --- | --- | --- | --- | --- | --- |
|  | **Odds ratio**  **(95% CI)** | **p-value** |  | **Odds ratio**  **(95% CI)** | **p-value** |
|  |  |  |  |  |  |
| ***All patients*** | 2.78 (1.53 to 5.04) | **<0.001** |  | 2.08 (1.01 to 4.26) | **0.046** |
|  |  |  |  |  |  |
| ***Age*** |  |  |  |  |  |
| >50 (n = 156) | 2.76 (1.24 to 6.11) | **0.013** |  | 1.90 (0.64 to 5.61) | 0.244 |
| ≤50 (n = 139) | 2.88 (1.13 to 7.35) | **0.027** |  | 1.49 (0.45 to 4.90) | 0.508 |
|  |  |  |  |  |  |
| **Gender** |  |  |  |  |  |
| Male (n = 275) | 2.51 (1.37 to 4.62) | **0.003** |  | 1.97 (0.94 to 4.14) | 0.072 |
|  |  |  |  |  |  |
| **Preexisting CKD** |  |  |  |  |  |
| No (n = 276) | 3.17 (1.69 to 5.93) | **<0.001** |  | 2.15 (1.03 to 4.45) | **0.040** |

**Table S7.** Treatment effect in the unweighted and weighted study populations in different subgroups.
*CI: confidence interval; CKD: chronic kidney disease*

|  | *N* | **Alive and free of new-onset or worsening CKD**  **(n = 236)** | **Alive and with new-onset or worsening CKD**  **(n = 45)** | **p-value*** |
| --- | --- | --- | --- | --- |
|  |  |  |  |  |
| ***Demography and medical history*** |  |  |  |  |
| Age, years | *281* | 51 (41, 58) | 53 (43, 62) | 0.252 |
| Cancer | *281* | 9 (3.8%) | 1 (2.2%) | 1.000 |
| Baseline eGFR, ml/min/1.73 m² | *142* | 103.7 (91, 112) | 89.2 (58, 97) | **0.012** |
| ***Physiology*** |  |  |  |  |
| Heart rate, bpm | 276 | 99.0 (86, 110) | 104.0 (82, 120) | 0.328 |
| Systolic blood pressure, mmHg | 276 | 119.0 (102.8, 131.0) | 119.5 (103.0, 133.) | 0.790 |
| Temperature, °C | 266 | 37.3 (36.6, 38.2) | 37.2 (36.5, 37.8) | 0.094 |
| Glasgow coma scale < 15 | 281 | 8 (3.4%) | 1 (2.2%) | 1.000 |
| **Biology** |  |  |  |  |
| Arterial partial O2 pressure, mmHg | 191 | 85.0 (75.8, 97.3) | 88.0 (76.5, 101.0) | 0.607 |
| Urea nitrogen, mmol/L | 281 | 19.1 (14.3, 24.7) | 24.6 (18.4, 31.4) | **<0.001** |
| Potassium, mmol/L | 280 | 3.5 (3.2, 3.9) | 3.7 (3.3, 4.2) | 0.164 |
| Bicarbonates, mmol/L | 280 | 22.0 (20.0, 24.0) | 21.0 (19.0, 23.0) | 0.172 |
| Sodium, mmol/L | 281 | 133.0 (130.0, 136.0) | 133.0 (130.0, 135.0) | 0.884 |
| Total bilirubin, µmol/L | 279 | 117.5 (46.5, 205.0) | 154.0 (69.0, 285.0) | 0.087 |
| Leukocytes, 10^9^ cells/L | 280 | 10.4 (8.3, 14.0) | 11.2 (8.2, 14.1) | 0.802 |

**Table S8.** Association between new-onset or worsening CKD at one year and baseline characteristics included in the propensity score in univariate analysis.

*bpm: beats per minute; eGFR: estimated glomerular filtration rate*

**Wilcoxon’s rank sum test was used for continuous variables; Fisher’s exact test or Pearson’s Chi-squared test were used for categorical variables, as appropriate. P-values are not adjusted for multiple comparisons and should be considered exploratory.*

**SUPPLEMENTAL FIGURES**

**
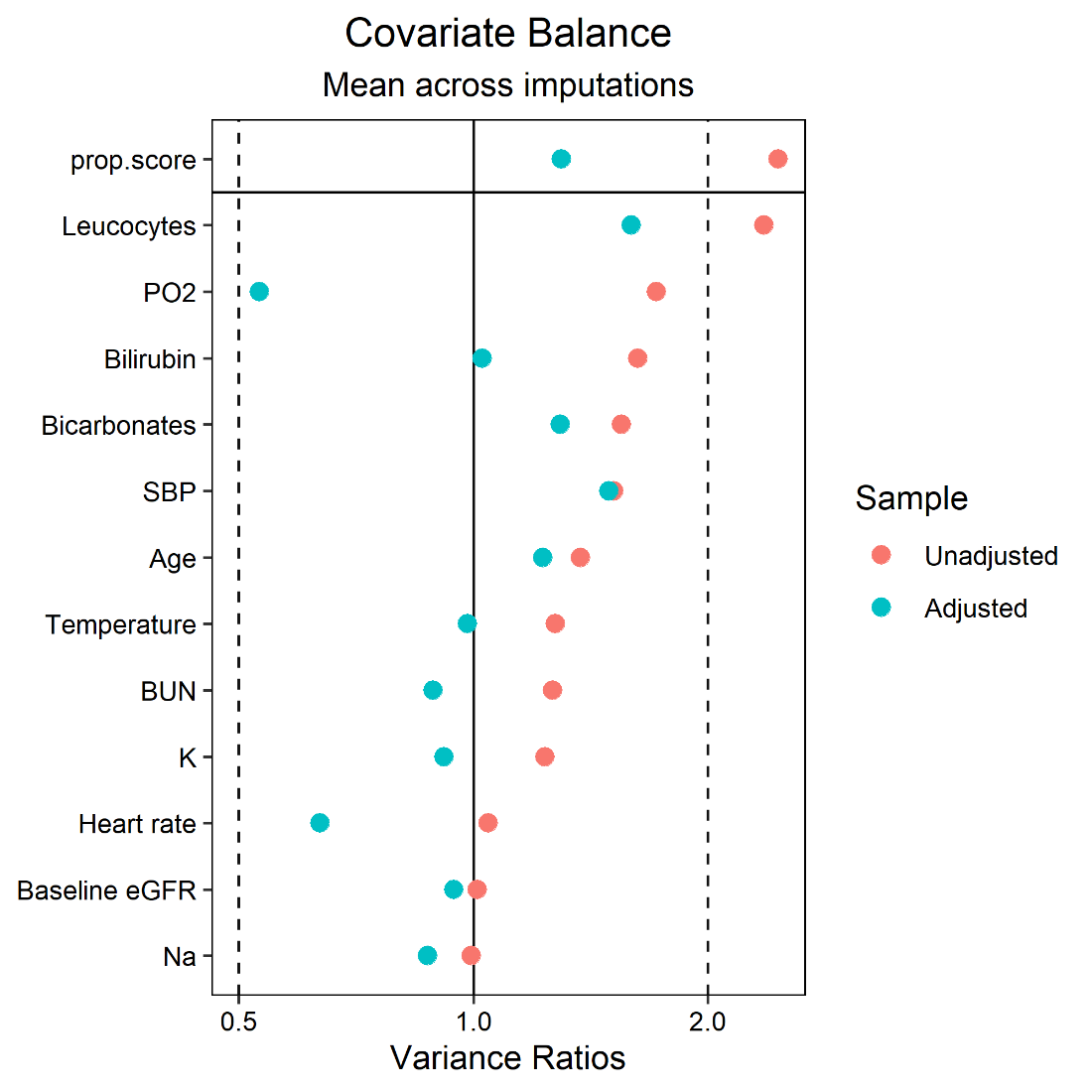
**

**Figure S1.** Variance ratios between groups for covariates of the propensity score in the unweighted and weighted populations.

*BUN: blood urea nitrogen; PO2: partial arterial oxygen pressure; K: potassium; eGFR: estimated glomerular filtration rate; Na: sodium; SBP: systolic blood pressure.*


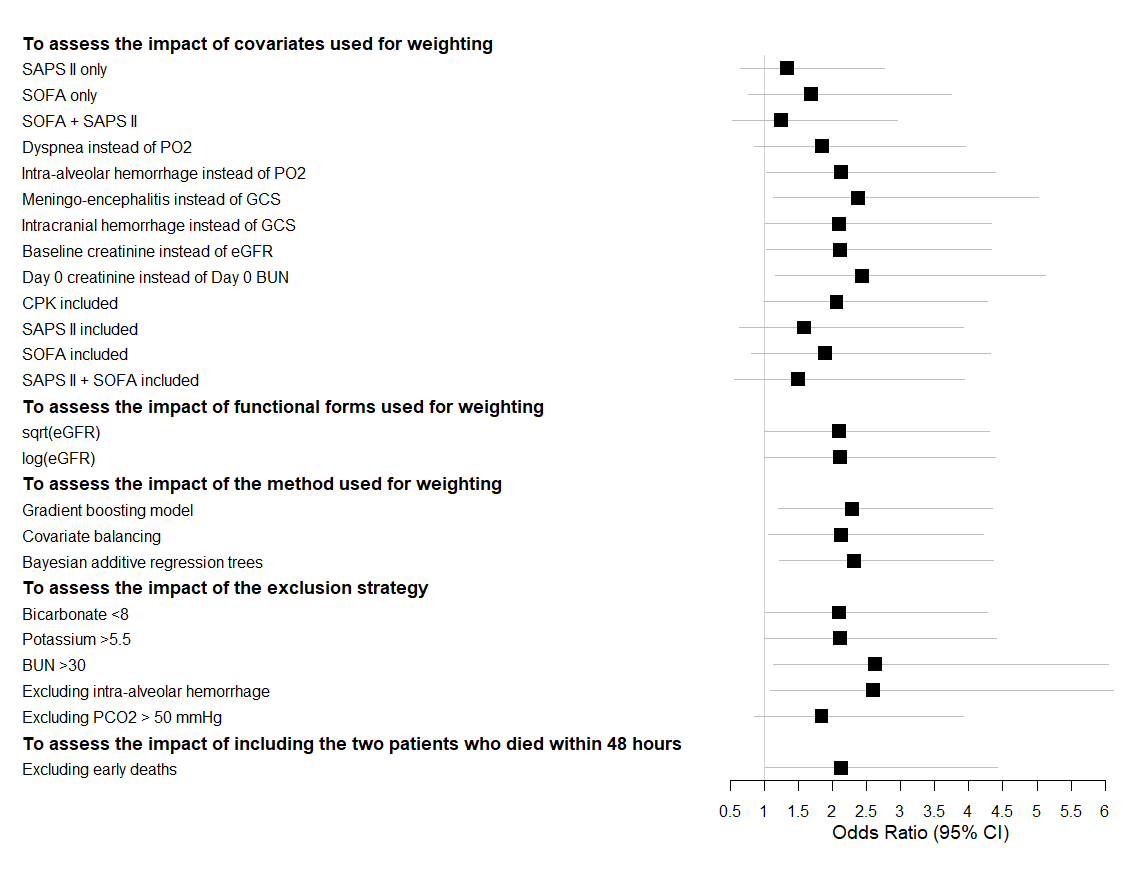
**Figure S2.** Treatment effect in the unweighted and weighted study populations in different sensitivity analyses.

*BUN: blood urea nitrogen: CI: confidence interval; CPK: creatinine phosphokinase; eGFR: estimated glomerular filtration rate: GCS: Glasgow coma scale; PCO2: partial pressure of carbon dioxide; PO2: partial pressure of oxygen; RRT: renal replacement therapy; SAPS II: Simplified Acute Physiology Score; SOFA: Sequential Organ Failure Assessment*

**SUPPLEMENTAL FILE**

STROBE Statement—checklist of items that should be included in reports of observational studies

|  | **Item No** | **Recommendation** | **Page  No** |
| --- | --- | --- | --- |
| **Title and abstract** | 1 | (*a*) Indicate the study’s design with a commonly used term in the title or the abstract | 1 |
|  |  | (*b*) Provide in the abstract an informative and balanced summary of what was done and what was found | 2 |
| **Introduction** | | | |
| Background/rationale | 2 | Explain the scientific background and rationale for the investigation being reported | 3 |
| Objectives | 3 | State specific objectives, including any prespecified hypotheses | 4 |
| **Methods** | | | |
| Study design | 4 | Present key elements of study design early in the paper | 5 |
| Setting | 5 | Describe the setting, locations, and relevant dates, including periods of recruitment, exposure, follow-up, and data collection | 5-8  Sup Methods |
| Participants | 6 | (*a*) *Cohort study*—Give the eligibility criteria, and the sources and methods of selection of participants. Describe methods of follow-up  *Case-control study*—Give the eligibility criteria, and the sources and methods of case ascertainment and control selection. Give the rationale for the choice of cases and controls  *Cross-sectional study*—Give the eligibility criteria, and the sources and methods of selection of participants | 5  Sup Methods |
|  |  | (*b*) *Cohort study*—For matched studies, give matching criteria and number of exposed and unexposed  *Case-control study*—For matched studies, give matching criteria and the number of controls per case | 5-8  Sup Methods |
| Variables | 7 | Clearly define all outcomes, exposures, predictors, potential confounders, and effect modifiers. Give diagnostic criteria, if applicable | 5-8  Sup Methods  Sup Tables S1 and S2 |
| Data sources/ measurement | 8* | For each variable of interest, give sources of data and details of methods of assessment (measurement). Describe comparability of assessment methods if there is more than one group | 5-6 |
| Bias | 9 | Describe any efforts to address potential sources of bias | 5-8  Sup Methods  Sup Tables S1-S3 |
| Study size | 10 | Explain how the study size was arrived at | 5-7 |
| Quantitative variables | 11 | Explain how quantitative variables were handled in the analyses. If applicable, describe which groupings were chosen and why | Sup Methods  Sup Table S6 |
| Statistical methods | 12 | (*a*) Describe all statistical methods, including those used to control for confounding | Sup Methods  Sup Tables S1 and S2 |
|  |  | (*b*) Describe any methods used to examine subgroups and interactions | 7-8  Sup Methods  Sup Table S6 |
|  |  | (*c*) Explain how missing data were addressed | 7  Sup Methods |
|  |  | (*d*) *Cohort study*—If applicable, explain how loss to follow-up was addressed  *Case-control study*—If applicable, explain how matching of cases and controls was addressed  *Cross-sectional study*—If applicable, describe analytical methods taking account of sampling strategy | Sup Methods  Sup Table S1 |
|  |  | (*e*) Describe any sensitivity analyses | 7-8  Sup Methods  Sup Table S6 |

Continued on next page

| **Results** | | | |
| --- | --- | --- | --- |
| Participants | 13* | (a) Report numbers of individuals at each stage of study—e.g., numbers potentially eligible, examined for eligibility, confirmed eligible, included in the study, completing follow-up, and analyzed | 9  Fig 1 |
|  |  | (b) Give reasons for non-participation at each stage | Fig 1 |
|  |  | (c) Consider use of a flow diagram | Fig 1 |
| Descriptive data | 14* | (a) Give characteristics of study participants (e.g., demographic, clinical, social) and information on exposures and potential confounders | 9  Table 2  Tables S4-S5 |
|  |  | (b) Indicate number of participants with missing data for each variable of interest | Tables S4-S5 |
|  |  | © *Cohort study*—Summarize follow-up time (e.g., average and total amount) | 9-10  Table 2 |
| Outcome data | 15* | *Cohort study*—Report numbers of outcome events or summary measures over time | Table 2 |
|  |  | *Case-control study—*Report numbers in each exposure category, or summary measures of exposure |  |
|  |  | *Cross-sectional study—*Report numbers of outcome events or summary measures |  |
| Main results | 16 | (*a*) Give unadjusted estimates and, if applicable, confounder-adjusted estimates and their precision (e.g., 95% confidence interval). Make clear which confounders were adjusted for and why they were included | Table 2 |
|  |  | (*b*) Report category boundaries when continuous variables were categorized | NA |
|  |  | (*c*) If relevant, consider translating estimates of relative risk into absolute risk for a meaningful time period | NA |
| Other analyses | 17 | Report other analyses done—e.g., analyses of subgroups and interactions, and sensitivity analyses | 9-11  Tables S6-S8  Fig S1 |
| **Discussion** | | | |
| Key results | 18 | Summarize key results with reference to study objectives | 12 |
| Limitations | 19 | Discuss limitations of the study, taking into account sources of potential bias or imprecision. Discuss both direction and magnitude of any potential bias | 12-15 |
| Interpretation | 20 | Give a cautious overall interpretation of results considering objectives, limitations, multiplicity of analyses, results from similar studies, and other relevant evidence | 12-15 |
| Generalizability | 21 | Discuss the generalizability (external validity) of the study results | 12-14 |
| **Other information** | | | |
| Funding | 22 | Give the source of funding and the role of the funders for the present study and, if applicable, for the original study on which the present article is based | 16 |

*Give information separately for cases and controls in case-control studies and, if applicable, for exposed and unexposed groups in cohort and cross-sectional studies.

**Note:** An Explanation and Elaboration article discusses each checklist item and gives methodological background and published examples of transparent reporting. The STROBE checklist is best used in conjunction with this article (freely available on the Web sites of PLoS Medicine at http://www.plosmedicine.org/, Annals of Internal Medicine at http://www.annals.org/, and Epidemiology at http://www.epidem.com/). Information on the STROBE Initiative is available at www.strobe-statement.org.
